# Personalized Connectome-Based Modeling in Patients with Semi-Acute Phase TBI: Relationship to Acute Neuroimaging and 6 Month Follow-Up

**DOI:** 10.1523/ENEURO.0075-21.2022

**Published:** 2022-02-15

**Authors:** Tyler Good, Michael Schirner, Kelly Shen, Petra Ritter, Pratik Mukherjee, Brian Levine, Anthony Randal McIntosh

**Affiliations:** 1 Rotman Research Institute, Baycrest Health Sciences, Toronto, Ontario M6A 2E1, Canada; 2 University of Toronto, Toronto, Ontario M5S 1A1, Canada; 3 Department of Neurology, Charité—Universitätsmedizin Berlin, corporate member of Freie Universität Berlin, and Humboldt-Universität zu Berlin, 10117 Berlin, Germany; 4 Berlin Institute of Health, Charité—Universitätsmedizin Berlin, 10178 Berlin, Germany; 5 Bernstein Center for Computational Neuroscience, Bernstein Focus State Dependencies of Learning, 10115 Berlin, Germany; 6 Einstein Center for Neurosciences Berlin, D-10117 Berlin, Germany; 7 Einstein Center Digital Future, 10117 Berlin, Germany; 8 Department of Radiology & Biomedical Imaging, University of California, San Francisco, San Francisco, California 94143-0628; 9 Brain and Spinal Cord Injury Center, Zuckerberg San Francisco General Hospital and Trauma Center, San Francisco, California 94143-0350; 10 Department of Bioengineering and Therapeutic Sciences, University of California, San Francisco, San Francisco, California 94158

**Keywords:** diffusion-weighted MRI, functional connectivity, functional MRI, netowrk modeling, structural connectivity, traumatic brain injury

## Abstract

Following traumatic brain injury (TBI), cognitive impairments manifest through interactions between microscopic and macroscopic changes. On the microscale, a neurometabolic cascade alters neurotransmission, while on the macroscale diffuse axonal injury impacts the integrity of long-range connections. Large-scale brain network modeling allows us to make predictions across these spatial scales by integrating neuroimaging data with biophysically based models to investigate how microscale changes invisible to conventional neuroimaging influence large-scale brain dynamics. To this end, we analyzed structural and functional neuroimaging data from a well characterized sample of 44 adult TBI patients recruited from a regional trauma center, scanned at 1–2 weeks postinjury, and with follow-up behavioral outcome assessed 6 months later. Thirty-six age-matched healthy adults served as comparison participants. Using The Virtual Brain, we fit simulations of whole-brain resting-state functional MRI to the empirical static and dynamic functional connectivity of each participant. Multivariate partial least squares (PLS) analysis showed that patients with acute traumatic intracranial lesions had lower cortical regional inhibitory connection strengths than comparison participants, while patients without acute lesions did not differ from the comparison group. Further multivariate PLS analyses found correlations between lower semiacute regional inhibitory connection strengths and more symptoms and lower cognitive performance at a 6 month follow-up. Critically, patients without acute lesions drove this relationship, suggesting clinical relevance of regional inhibitory connection strengths even when traumatic intracranial lesions were not present. Our results suggest that large-scale connectome-based models may be sensitive to pathophysiological changes in semi-acute phase TBI patients and predictive of their chronic outcomes.

## Significance Statement

The variability of clinical outcomes following mild to moderate traumatic brain injury (TBI) is underscored by complex pathophysiological mechanisms that take effect across spatial scales. We used the neuroinformatics platform, The Virtual Brain, to model individualized brain activity and make inferences across these spatial scales. Specifically, this approach allowed us to link macroscopic brain dynamics with mesoscopic biophysical parameters, distinguishing semiacute mild to moderate TBI patients from comparison participants and predicting the long-term recovery of these patients. Our results demonstrate the sensitivity of our large-scale brain model to pathophysiological changes following TBI and illustrates how computational modeling may be used to advance understanding of chronic TBI outcome.

## Introduction

Chronic clinical outcomes following traumatic brain injury (TBI) are heterogeneous (Dabek and Caban, 2016; Si et al., 2018). Classifying patients based on the presence of pathoanatomic features on computerized tomography (CT) and/or magnetic resonance imaging (MRI) is a useful way to stratify the variance of TBI patients (Iverson et al., 2012; McMahon et al., 2014; Yuh et al., 2014; Palacios et al., 2017). However, even within these patient subgroups significant variability in clinical outcomes and cognitive performance can be observed (Iverson et al., 2012; Palacios et al., 2017). The sources of this variability are diverse (Kenzie et al., 2018) and may include forms of pathology not visible with conventional neuroimaging.

TBI has been described as a multiscale system deficit, with cognitive impairments manifesting through interactions between microscopic and macroscopic changes (Kenzie et al., 2018). On the macroscale, TBI is associated with decreased integrity of white matter (WM) pathways and an imbalance and inefficiency of functional networks (Hayes et al., 2016). Studies using diffusion-weighted MRI (dwMRI) have consistently detected decreases in fractional anisotropy (FA; Niogi and Mukherjee, 2010; Shenton et al., 2012; Douglas et al., 2015; Filley and Kelly, 2018) that correlate with cognition (Wallace et al., 2018; Palacios et al., 2020) and, if assessed in the semiacute phase, long-term clinical outcomes (Yuh et al., 2014). Functional connections are also sensitive to TBI, with alterations observed in multiple intrinsic connectivity networks (for review, see Sharp et al., 2014; Hayes et al., 2016). Early-phase functional connectivity (FC) between and within networks may be predictive of long-term symptom severity (Palacios et al., 2017; Madhavan et al., 2019), while changes to FC dynamics (FCD) have been noted in the acute phase (Hou et al., 2019) and semiacute phase (Mayer et al., 2015; Vergara et al., 2018) following TBI.

On the microscale, TBI causes a neurometabolic cascade that alters neurotransmission and can have long-lasting effects (Giza and Hovda, 2015). The initial injury causes a sudden imbalance in glutamatergic and GABAergic neurotransmitter levels, as well as NMDA receptor malfunction (Giza and Hovda, 2015). Magnetic resonance spectroscopy (MRS) is capable of detecting changes to neuromodulatory concentrations *in vivo*. A recent meta-analysis found evidence for elevated glutamate concentrations in adult patients after a single mild TBI (mTBI) in the acute and subacute phases (Eisele et al., 2020). Furthermore, some studies have shown that these elevated glutamate concentrations are predictive of long-term outcomes (Shutter et al., 2004).

**Figure 1.**
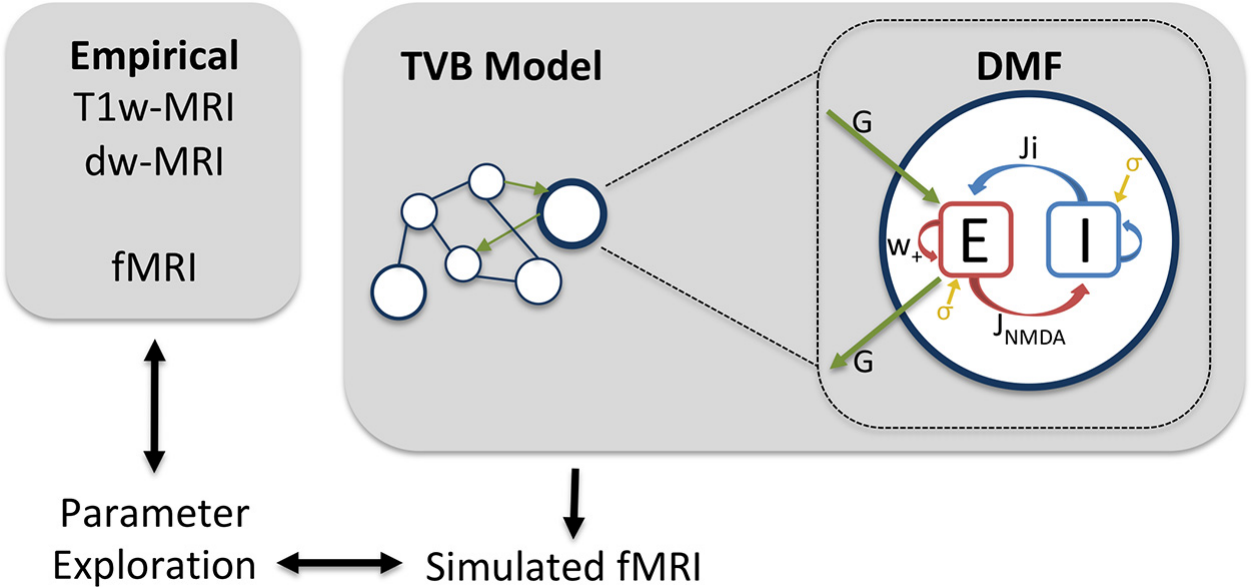
The Virtual Brain workflow. Structural and functional connectomes were created from each subject’s dwMRI and fMRI data, respectively. Each subject’s unique structural connectome constrained their personal brain simulation, wherein local dynamics were represented by the dynamic mean field (DMF) model (Eqs. 1–6; Deco et al., 2014a,b). The simulated local synaptic gating potentials were then fed through the Balloon–Windkessel hemodynamic model, producing simulated fMRI time series. Each subject’s simulated fMRI time series was fitted to their functional connectome through parameter space exploration. The resulting subject-specific parameters were used in later analyses. *E*, Excitatory neural population; *I*, inhibitory neural population; *w*_+_, recurrent potential; *J*_NMDA_, excitatory connection strength.

Connectome-based brain network modeling provides a novel perspective to TBI by allowing access to local and global parameters related to both micro-level neuromodulatory changes and macro-level connectivity changes. We used the neuroinformatics platform The Virtual Brain (TVB) to simulate whole-brain dynamics composed of interacting neural population models (Fig. 1; Ritter et al., 2013; Sanz Leon et al., 2013). Our model simulated brain areas as excitatory and inhibitory neural populations connected via GABA and NMDA synapses (Deco et al., 2014b; Schirner et al., 2018). Personalized simulations were constrained by each subject’s structural connectome and fitted to their static and dynamic functional connectomes, which were computed from dwMRI and resting-state fMRI (rsfMRI; Schirner et al., 2015). The parameter-fitting procedure tuned the parameters global coupling (*G*) and regional inhibitory connection strengths to yield FC predictions for each subject. Global coupling is a scaling factor related to the level of integration/segregation in the system (Deco et al., 2015), while regional inhibitory connection strength indicates the level of inhibitory influence at each brain region. These model parameters have been used to describe healthy neural dynamics (Jirsa et al., 2010; Roy et al., 2014) and those of clinical populations (Falcon et al., 2016; Jirsa et al., 2017; Aerts et al., 2018; Zimmermann et al., 2018).

In the present exploratory study, we considered a well characterized sample of TBI patients and healthy comparison participants. Previous studies of this sample have described group differences and predictive correlations between semi-acute phase neuroimaging data and 6 month behavioral outcome measures using fractional anisotropy (Yuh et al., 2014) and functional connectivity (Palacios et al., 2017). In the present work, we integrated these modalities with personalized TVB simulations, which allowed us to consider the role of local neural dynamics in patient outcomes. We examined group differences as well as predictive correlations between semiacute local brain dynamics and clinical outcomes at a 6 month follow-up. Our analyses considered the following two patient subgroups: CT/MRI positive, defined as patients with any signs of traumatic intracranial lesions on day-of-injury CT scan or semiacute MRI; and CT/MRI negative, defined as patients without any such abnormality.

## Materials and Methods

### Participants

Forty-four patients and 36 comparison participants were acquired from the pilot phase of the Transforming Research and Clinical Knowledge in Traumatic Brain Injury project (Track-TBI Pilot; Yue et al., 2013). Patients were collected from convenience sampling at the acute-care, level I trauma center in San Francisco General Hospital. The inclusion criteria included a CT scan to assess for evidence of acute TBI within 24 h of injury, Glasgow Coma Scale score of 13–15 [on emergency department (ED) arrival], loss of consciousness (LOC) <30 min, post-traumatic amnesia duration of <24 h, and age 18–55 years (inclusive). Patients with a previous significant TBI (LOC for >5min) were also excluded. Overlapping patient and comparison participant samples have been described in detail in two previous publications (Yuh et al., 2014; Palacios et al., 2017). Eight patients did not complete the clinical and cognitive assessment at the 6 month follow-up and were therefore removed from analyses using these data (*n*_patient_ = 36 for 6 month clinical/cognitive data).

Each patient’s head CT on ED presentation and semiacute brain MRI (5–18 d postinjury) was characterized using the TBI common data elements criteria (Yue et al., 2013). Each CT and MRI was anonymized and reviewed by a board-certified neuroradiologist blinded to the data. The TBI patients were divided into the following two subgroups: (1) CT/MRI positive (*n* = 14; age: mean = 39 years; SD = 13.7), defined as patients with any acute traumatic intracranial lesion (epidural hematoma, subdural hematoma, subarachnoid hemorrhage, contusion, or evidence of traumatic axonal injury) and/or depressed skull fracture on either CT or MRI; and (2) CT/MRI negative (*n* = 30; age: mean = 31 years; SD = 9.0), defined as patients without any such abnormality on either CT or MRI. There were no large lesions expected to adversely affect the dwMRI or fMRI results. Patient characteristics are presented in Table 1. We note that all of the TBI patients meet the definition of mTBI by some standards (Yue et al., 2013), though the patients with positive CT findings would be considered moderate by other criteria (Krainin et al., 2011).

**Table 1:** Patient characteristics

Scale	Subscale	CT/MRI-positive (*n* = 14)	CT/MRI negative (*n* = 30)	Comparison participants (*n* = 36)	Analysis for group difference
Age (years)		39.9 (13.8)	31.2 (9.0)	26.6 (7.7)	*F*_(2,79)_ = 9.7, *p* = 0.0001
Gender		9 male; 5 female	18 male; 12 female	25 male; 11 female	χ(2)^2^ = 0.65, *p* = 0.72
Race		9 white, 2 More than one race; 1 African American or African; 1 Asian; 1 Hawaiian or Pacific Islander	23 white; 3 Asian; 2 Hawaiian or Pacific Islander; 1 African American or African; 1 more than one race	Unknown	
Education		14.6 (2.1)	14.6 (3.1)	Unknown	*t*_(42)_ = −0.08, *p* = 0.93
Glasgow Coma Scale		14.6 (0.63)	14.9 (0.43)	NA	*t*_(42)_ = 1.4, *p* = 0.18
Loss of consciousness		8 none; 6 < 0.5 h	12 none; 18 < 0.5 h	NA	χ(1)^2^ = 0.55, *p* = 0.46
Post-traumatic amnesia		5 none; 4 < 0.5 h; 5 0.5–24 h	14 None; 15 < 0.5 h; 1 0.5-24 h	NA	χ(1)^2^ = 8.6, *p* = 0.01
		*n* = 11	*n* = 27	NA	
Glasgow Outcome Scale Extended	6 month	6.8 (0.98)	7.0 (0.94)	NA	*t*_(36)_ = 0.64, *p* = 0.52
Brief Symptom Inventory	Anxiety	54.9 (7.7)	53.6 (10.6)	NA	*t*_(36)_ = −0.37, *p* = 0.71
	Depression	54.2 (10.2)	52.6 (10.6)	NA	*t*_(36)_ = −0.42, *p* = 0.67
	Somatic	55.8 (7.9)	53.0 (9.7)	NA	*t*_(36)_ = −0.86, *p* = 0.39
	Global Severity Index	57.0 (7.3)	53.6 (10.8)	NA	*t*_(36)_ = −0.97, *p* = 0.34
		*n* = 11	*n* = 26	NA	
Satisfaction with Life Score		19.1 (7.6)	22.6 (5.9)	NA	*t*_(35)_ = 1.5, *p* = 0.14
		*n* = 11	*n* = 25	NA	
Trail Making Test	Part A	30.7 (9.7)	28.3 (10.6)	NA	*t*_(35)_ = −0.65, *p* = 0.52
	Part B	69.8 (25.0)	76.4 (65.2)	NA	*t*_(35)_ = 0.32, *p* = 0.75
Wechsler Adult Intelligence Scale	Processing Speed	109.2 (16.3)	106.5 (14.6)	NA	*t*_(35)_ = −0.49, *p* = 0.62
California Verbal Learning Test		55.5 (9.8)	55.5 (9.2)	NA	*t*_(35)_ = 0.06, *p* = 0.98

The following statistics are reported: one-way ANOVA (age), χ^2^ test of independence (gender, loss of consciousness, post-traumatic amnesia), independent-samples *t* test [Education, Glasgow Coma Scale, Glasgow Outcome Scale Extended (6 month), Brief Symptom Inventory, Satisfaction with Life Score, Trail Making Test Part A and B, Wechsler Adult Intelligence Scale, and California Verbal Learning Test. NA, Not applicable. Data are mean (SD), unless otherwise indicated.

### Outcome measures

The outcome measures included the Extended Glasgow Outcome Scale (GOS-E) at 6 months postinjury performed through structured interviews with each participant by research assistants trained to uniformly assess the GOS-E. A trained neuropsychologist also administered the following behavioral and cognitive tests at 6 months after injury: Trail Making Tests (TMTs) Parts A and B, Wechsler Adult Intelligence Scale (WAIS), fourth edition; Satisfaction with Life Scale (SWLS; Diener et al., 1985); Brief Symptom Inventory (BSI) 18 (Derogatis and Melisaratos, 1983); and California Verbal Learning Test (CVLT), second edition (Delis et al., 2000). Higher scores on the GOS-E, SWLS, WAIS, and CVLT were coded such that higher scores indicate better outcome, while scores on the BSI and TMT are coded such that higher scores indicate poorer outcome.

### Principal component analysis

Principal component analysis (PCA) was used to expose the latent structure within the collection of outcome measures and to reduce the dimensionality of the dataset. Data were transformed if significantly skewed (*p *<* *0.05) using square root or log10 to improve the correlation structure. The point of inflection on a scree plot was used to identify the number of components to keep. After extracting this number of factors, a promax rotation was performed using the “principal” function in R. The correlation between factors was *r* = 0.33, exceeding 0.32 and therefore indicating >10% overlap in variance between factors, warranting an oblique rotation (Tabachnick and Fidell, 2001). As such, we used the two-factor solution with a promax rotation for later analyses. Factors were designated as (1) TBI Symptoms, and (2) Age and Cognition based on the primary symptoms, cognitive functions, and characteristics assessed by the variables that strongly contributed to each (loading, >0.3; see Table 5). Factor scores for each participant represent the degree to which they express the factor. Higher factor scores on the TBI Symptoms factor indicate poorer outcome, while higher factor scores on the Age and Cognition factor imply older age and lower performance (Tables 2-Tables 4).

**Table 2:** MRI radiologic findings of the CT/MRI-positive TBI group

1	4 contusions, 1 shear (MRI)
2	2 shear (MRI)
3	1 shear (MRI)
5	2 shear (MRI)
5	4 contusions (MRI) 1 intracranial lesions, 1 skull fracture, 1 subdural hematoma, 1 contusion, 1 brain swelling (CT)
6	4 contusions, 3 shears (MRI)
7	2 shear (MRI)
8	1 skull fracture (CT)
9	1 contusion, 2 shear (MRI) 1 intracranial lesions, 1 sub arachnoid hemorrhage, 1 contusion
10	1 subdural hematoma, 2 contusions (MRI) 1 intracranial lesions, 1 subarachnoid hemorrhage, 1 contusion
11	1 subdural hematoma, 2 contusions, 2 shear (MRI) 1 intracranial lesions, 1 skull fracture, 1 subdural hematoma, 1 subarachnoid hematoma
12	1 intracranial lesions, 1 subarachnoid hematoma (CT)
13	2 shear (MRI)
14	1 shear, 1 deep shear (MRI)

**Table 3:** Cortical and subcortical regions from the regional map parcellation from Kötter and Wanke (2005)

Index		Region
Right	Left	
1	49	Primary auditory cortex
2	50	Secondary auditory cortex
3	51	Amygdala
4	52	Anterior cingulate cortex
5	53	Posterior cingulate cortex
6	54	Retrosplenial cingulate cortex
7	55	Subgenual cingulate cortex
8	56	Frontal eye field
9	57	Gustatory cortex
10	58	Hippocampus
11	59	Anterior insula
12	60	Posterior insula
13	61	Primary motor cortex
14	62	Inferior parietal cortex
15	63	Intraparietal cortex
16	64	Medial parietal cortex
17	65	Superior parietal cortex
18	66	Centrolateral prefrontal cortex
19	67	Dorsolateral prefrontal cortex
20	68	Dorsomedial prefrontal cortex
21	69	Medial prefrontal cortex
22	70	Orbitoinferior prefrontal cortex
23	71	Orbitolateral prefrontal cortex
24	72	Orbitomedial prefrontal cortex
25	73	Prefrontal polar cortex
26	74	Ventrolateral prefrontal cortex
27	75	Parahippocampal cortex
28	76	Dorsolateral premotor cortex
29	77	Medial premotor cortex
30	78	Ventrolateral premotor cortex
31	79	Primary somatosensory cortex
32	80	Secondary somatosensory cortex
33	81	Central temporal cortex
34	82	Inferior temporal cortex
35	83	Temporal polar cortex
36	84	Superior temporal cortex
37	85	Ventral temporal cortex
38	86	Visual area 1 (primary visual cortex)
39	87	Visual area 2 (secondary visual cortex)
40	88	Anterior visual area, dorsal part
41	89	Anterior visual area, ventral part
42	90	Thalamic ROI with major frontal connections
43	91	Thalamic ROI with major temporal connections
44	92	Thalamic ROI with major occipitoparietal connections
45	93	Caudate nucleus
46	94	Putamen
47	95	Pallidum
48	96	Accumbens nucleus

**Table 4:** TVB Model parameters

Parameter	Value (no. of steps)	Description
*G*	1.4–2.8 (50)	Scaling factor for inter-region (global) excitatory coupling
Noise ( σ)	0.001	Amplitude of noise kernel
Conduction velocity (m/s)	6	Speed of inter-region (global) signal transmission
w_+_	1.4	Excitatory recurrent potential
J_GABA_ (nA)	1.0*	Local feedback inhibitory synaptic coupling
J_NMDA_ (nA)	0.15	Local excitatory coupling
Time steps (ms)	600,000	Simulation duration
fMRI TR (ms)	2000	Simulation TR

*J_GABA_ values were initialized at 1.0 and adjusted iteratively by the FIC tuning algorithm during each simulation.

**Table 5:** Factor loadings for 6 month outcome variables

Scale	Subscale	TBI symptoms	Age and cognition
Glasgow Outcome Scale Extended	6 month	**−0.73**	−0.033
Brief Symptom Inventory	Somatic	**0.71**	0.16
	Depression	**0.86**	−0.075
	Anxiety	**0.87**	−0.032
	Global severity index	**0.99**	−0.043
Satisfaction with Life Scale		**−0.83**	0.29
Education		**−0.33**	**−0.51**
Age		−0.036	**0.42**
Trail Making Test	Part A	0.19	**0.62**
	Part B	−0.085	**0.89**
Wechsler Adult Intelligence Scale	Processing speed	0.25	**−0.92**
California Verbal Learning Test		−0.10	**−0.31**
Percentage covariance		37%	22%

Loadings >0.3 are shown in bold to assist interpretation. BSI and TMT scales are reverse coded such that higher scores indicate more symptoms or poorer performance.

### Imaging procedure

MRIs were acquired on a scanner (SIGNA EXCITE 3 T MRI, GE Healthcare) equipped with an eight-channel phased array head radio frequency coil. The following conventional 3 T MRI sequences were performed: (1) axial three-dimensional inversion recovery fast spoiled gradient recalled echo T1-weighted images [echo time (TE) = 1.5 ms; response time (TR) = 6.3 ms; inversion time (TI) = 400 ms; flip angle, 15°] with 230 mm field of view (FOV) and 156 contiguous partitions (1.0 mm) at a 256 · 256 matrix; (2) axial T2-weighted fluid-attenuated inversion recovery images (TE = 126 ms; TR= 10 s; TI = 2200 ms) with 220 mm FOV, and 47–48 contiguous slices (3.0 mm) at a 256 · 256 matrix; and (3) axial magnetization-prepared gradient echo T2*-weighted images (TE = 15 ms; TR = 500 ms; flip angle 20°) with a 220 · 170 mm FOV and 47–48 contiguous slices (3.0 mm) at a 256 · 192 matrix. A 7 min rsfMRI single-shot gradient-echo echoplanar imaging sequence was acquired (TR = 2000 ms; TE = 28 ms; flip angle = 90° gradient; FOV = 220 mm; voxel size = 3.4 · 3.4 · 4.0 mm). The subjects were asked to close their eyes, relax, not focus their attention on anything specific, and not fall asleep. Whole-brain diffusion tensor imaging (DTI) was performed with a multislice single-shot, spin-echo echoplanar pulse sequence (TE = 63 ms; TR = 14 s) using 55 diffusion-encoding directions, isotropically distributed over the surface of a sphere with electrostatic repulsion, acquired at *b* = 1000 s/mm^2^, seven acquisitions at *b* = 0 s/mm^2^, 72 interleaved slices of 1.8 mm thickness each with no gap between slices, a 128 · 128 matrix, and an FOV of 230 · 230 mm. For DTI, parallel imaging was used using the array spatial sensitivity encoding technique with an acceleration factor of 2. The MRI scanner and the scanning protocol used were the same for the patient and comparison groups.

### Parcellation scheme

The 96-region of interest (ROI) regional map (RM-96) parcellation (Kötter and Wanke, 2005) was used for construction of the structural connectivity (SC) and functional connectivity matrices. The RM-96 parcellation has 82 cortical and 14 subcortical ROIs (Bezgin et al., 2017). It has been used previously in TVB models (Ritter et al., 2013; Shen et al., 2019a) and was developed to harmonize cytoarchitectonic, topographic and functional definitions of brain regions across primate species (Kötter and Wanke, 2005), which is an advantage for network modeling work that integrates structural and functional neuroimaging data.

### dwMRI preprocessing and tractography

Preprocessing of dwMRI data, and subsequent tractography was completed using a Python implementation of a previously reported procedure (Shen et al., 2019b). Eddy current-induced distortions were corrected for using the FSL “eddy_correct” command, and the diffusion gradient vectors rotated accordingly. The MNI152_T1_1mm standard brain included with FSL was then registered to each subject’s T1-weighted image using a nonlinear registration conducted with Advanced Normalization Tools (ANTs). Warps produced in this step were used to map the 96-RM parcellation (ANTs: Avants et al., 2011; RM-96 parcellation: Bezgin et al., 2017) onto each subject’s T1-weighted image using the ANTs “WarpImageMultiTransform” executable and a nearest neighbor interpolation. The FSL FLIRT function was used to register subject T1-weighted images to dwMRI space. Seed and target ROI masks were defined as the WM voxels adjacent to each gray matter (GM) ROI within each region of the RM-96 parcellation scheme. An exclusion mask for each seed mask was also created using the GM voxels adjacent to the seed mask. For intrahemispheric tracking, exclusion masks of the opposite hemisphere were also used. Diffusion tensor models were fitted at each voxel by FSL “dtifit,” and then a probabilistic diffusion model was fit using FSL “bedpostX.” Probabilistic tractography was performed between all ROIs of the RM-96 parcellation scheme using the FSL “probtrackx2” function. The parameters used for tracking were as follows: 5000 seeds per voxel, 2000 steps, 0.5 mm step length, termination of paths that loop back on themselves, and rejection of paths that pass through an exclusion mask. The curvature threshold was set to 0.2.

Two SC matrices were constructed from the tractography results representing weights and lengths of connections, respectively. Weights were generated by taking the number of streamlines detected between each ROI pair and dividing it by the total number of streamlines that were successfully sent from the seed mask. In this way, they were corrected for the number of voxels in each seed ROI. The length of each connection, defined in millimeters, was obtained by taking the median length of all connecting streamlines for each region pair. To obtain tract lengths using FSL probtrackx2, tractography was run with and without the distance correction, and dividing the results gave an estimate of the lengths of each streamline. As tractography cannot provide information on directionality of connections, matrices were symmetrized such that the weights between areas A to B and B to A were averaged. To account for numerous false positives that are known to result from probabilistic tractography (Shen et al., 2019b), any connections not present in at least 50% of the comparison subjects were set to zero (Roberts et al., 2017).

### Resting-state fMRI

Resting-state fMRI preprocessing was done using the FMRIB FEAT toolbox. The following steps were performed: (1) motion correction with MCFLIRT (Jenkinson et al., 2002), (2) slice timing correction, (3) spatial smoothing (5 mm), and (4) registration to anatomic volume. A nuisance regression removed signals from CSF, white matter, and six motion parameters. Global signal regression was not performed. Last, a high-pass filter (100 s) was applied.

A weighted average time series was calculated for every ROI, such that each voxel was weighted according to the probability it was within a given ROI (all voxels summed to one), with voxels more central in the ROI being favored (Shen et al., 2012). This approach aimed to minimize partial volume bias. An FC matrix was calculated for each subject by finding the pairwise Pearson’s correlation coefficient between the weighted average time series of each ROI pair. To characterize the dynamics of the resting fluctuations, we calculated FCD matrices for each subject. The 7 min resting-state scan was split into 92 windows of 30 s overlapping by 4 s. We calculated the FC matrix for each time window centered at time *t*, generating a time series of FC matrices, FC(*t*). The FCD matrix is a *t* by *t* matrix with element (*t*1, *t*2) calculated as the Pearson’s correlation of the upper triangle of the FC matrices FC(*t*1) and FC(*t*2). This method of characterizing resting fluctuations with FCD has been used previously (Deco et al., 2017).

No participants were removed because of head motion during the resting-state fMRI scan (absolute head motion at a single time point was not more than 3.5 mm, and relative head motion did not exceed 2.5 mm for any participants. A one-way ANOVA showed the comparison participants (mean* *=* *0.27, SD* *=* *0.28), CT/MRI-positive patients (mean =* *0.25, SD* *=* *0.20), and CT/MRI-negative patients (mean = 0.23, SD* *=* *0.17) did not differ in mean absolute head motion (*F*_(2,77)_ = 0.22, *p* = 0.801). Similarly the comparison participants (mean* *=* *0.06, SD* *=* *0.03), CT/MRI-positive (mean* *= 0.07, SD* *=* *0.02), and CT/MRI-negative patients (mean* *=* *0.10, SD* *=* *0.09) did not differ in mean relative head motion (*F*_(2,77)_ = 2.00, *p* = 0.14).

### The Virtual Brain

We simulated fMRI time series for each subject. Simulations were constrained by each subject’s empirical SC and fitted to their FC and FCD matrices. The process for each subject may be summarized as follows: (1) SC matrices (weights and tract lengths) are input to fast_tvb (https://github.com/BrainModes/fast_tvb, https://hub.docker.com/r/thevirtualbrain/fast_tvb); (2) local and global parameters are chosen for the model; (3) fMRI is simulated based on local and global parameters and is constrained by individual SC; (4) individual fMRI simulations are optimized by rerunning simulations with different local and/or global parameter values that yielded the best fit; and (5) the best-fitting local and global parameters are used for group comparisons and to account for individual variability on our neuropsychological factors. The modeling process in TVB has been described more thoroughly previously (Ritter et al., 2013; Sanz Leon et al., 2013), and the mean field approximations used have been validated independently (Deco and Hugues, 2012). Readers may also refer to an excellent, more general review of dynamic models of large-scale brain activity (Breakspear, 2017).

### Dynamic mean field model

The dynamic mean field model represents each region of interest as a population of excitatory and inhibitory neurons coupled by excitatory NMDA synapses and inhibitory GABA synapses. The model is defined by a set of six stochastic nonlinear differential equations, modified slightly from those presented by Deco et al. (2014a,b), such that the global, inter-region connections incorporated time delays, as follows:

(1) $$I_{i}^{\left(E\right)}\left(t\right)=W_{E}I_{0}+w_{+}J_{\text{NMDA}}S_{i}^{\left(E\right)}(t)+GJ_{\text{NMDA}}\underset{j}{\sum }C_{ij}S_{j}^{\left(E\right)}\left(t-\frac{D_{ij}}{s}\right)-J_{i}S_{i}^{\left(I\right)}\left(\text{t}\right)$$

(2) $$I_{i}^{\left(I\right)}\left(t\right)=W_{I}I_{0}+J_{\text{NMDA}}S_{i}^{\left(E\right)}(t)-S_{i}^{\left(I\right)}(t)$$

(3) $$r_{i}^{\left(E\right)}\left(t\right)=\frac{a_{E}I_{i}^{\left(E\right)}\left(t\right)-b_{E}}{1-\text{exp}(-d_{E}\left(a_{E}I_{i}^{\left(E\right)}\left(t\right)-b_{E}\right))}$$

(4) $$r_{i}^{\left(I\right)}\left(t\right)=\frac{a_{I}I_{i}^{\left(I\right)}\left(t\right)-b_{I}}{1-\text{exp}(-d_{I}\left(a_{I}I_{i}^{\left(I\right)}\left(t\right)-b_{I}\right))}$$

(5) $$\frac{dS_{i}^{\left(E\right)}(t)}{dt}=\frac{-S_{i}^{\left(E\right)}\left(t\right)}{\tau_{E}}+\left(1-S_{i}^{\left(E\right)}\left(t\right)\right)\gamma r_{i}^{\left(E\right)}(t)+\sigma v_{i}\left(t\right)$$

(6) $$\frac{dS_{i}^{\left(I\right)}(t)}{dt}\left(t\right)=\frac{-S_{i}^{\left(I\right)}\left(t\right)}{\tau_{I}}+r_{i}^{\left(I\right)}(t)+\sigma v_{i}\left(t\right).$$

*I_i_*^(^*^E^*^)^ represents the input current to the excitatory population of region *i*, while *I_i_*^(^*^I^*^)^ denotes the input current to the inhibitory population at that region. Equations 3 and 4 convert input current to firing rates, *r_i_*^(^*^E/I^*^)^, for the excitatory, and inhibitory populations of region *i* respectively. Finally, the firing rate is used to calculate synaptic gating (*S_i_*^(^*^E^*^/^*^I^*^)^) of both the excitatory (*E*) and inhibitory (*I*) populations in Equations 5 and 6.

Input current to the excitatory population is defined by the following four sources: the overall effective external current ( $W_{E}I_{0})$, recurrent excitatory currents $\left[w_{+}J_{\text{NMDA}}S_{i}^{\left(E\right)}(t\right)$], recurrent inhibitory currents [ $J_{i}S_{i}^{\left(I\right)}(t)$], and excitatory currents from the excitatory populations of the other region [ $GJ_{\text{NMDA}}\underset{j}{\sum }C_{ij}S_{j}^{\left(E\right)}\left(t-\frac{D_{ij}}{s}\right)]$. Long-range (inter-region) connections between regions *i* and *j* are constrained by the connectivity weight *C_ij_*, where *C_ij_* is the (*i*, *j*)*-*th entry in the SC weights matrix. *G* strength scales the long-range connectivity weights (*C_ij_*). Time delays are incorporated through the division of the distance between regions *i* and *j* (*D_ij_*), by conduction velocity (*s*). The input current to the inhibitory population is defined by the following: external currents ( $W_{I}I_{0})$, recurrent excitatory currents $\left[J_{\text{NMDA}}S_{i}^{\left(E\right)}(t)\right]$, and recurrent inhibitory currents $\left[S_{i}^{\left(I\right)}(t)\right]$. Notably, noise (σ) is added in Equations 5 and 6, where *v_i_* is uncorrelated standard Gaussian noise, with amplitude scaled by σ.

To maintain an average firing rate between 2 and 5 Hz feedback inhibition control (FIC) was applied (Deco et al., 2014a; Schirner et al., 2018). The FIC algorithm iteratively adjusts the inhibitory connection weights (*J_i_*) at each region so that each local excitatory population maintains an average firing rate of ∼3 Hz. By maintaining local excitation–inhibition balance in this way, we produce simulations that fit better with empirical fMRI and show more realistic firing rates (Deco et al., 2014a).

The differential equations were integrated with a step size of 0.1 ms. After starting a simulation, first the *J_i_* values were fitted using an automatic routine, and then 8 min and 20 s of fMRI were simulated to match the duration of the empirical fMRI time series. Eighty seconds of simulated fMRI data were removed to account for initial transients. Simulated synaptic activity was fed through the Balloon–Windkessel hemodynamic model producing simulated fMRI data (Friston et al., 2000). All simulations were performed using an implementation of the dynamic mean field model in C (Schirner et al., 2018; code available as follows: https://github.com/BrainModes/fast_tvb).

### Parameter space exploration

Subject-specific parameter space explorations found the best-fitting value of global coupling to maximize the fit of their simulation to their empirical FC and FCD. To avoid overfitting, we used two features to define goodness-of-fit. Our first feature was a maximal uncentered Pearson correlation of the upper triangle of each subject’s empirical and simulated FC matrices. The second was the minimal Kolmogorov–Smirnov distance between the distributions formed by the upper triangle of the simulated and empirical FCD matrices. The uncentered Pearson correlation takes into account the difference in the mean values of the FC matrices (Deco et al., 2014a), and fitting FCD matrix distributions with Kolmogorov–Smirnov distance has been used previously to effectively fit brain dynamics in network models (Deco et al., 2017). We explored 50 values of *G* for each subject, and, to account for the effect of intrasubject variability because of our stochastic model, we ran 20 iterations of the parameter space exploration (PSE) for each subject with randomized initial conditions, resulting in 1000 simulations being run for every subject. Regional inhibitory connection strengths were fitted for each of these simulations via the FIC tuning algorithm described in the previous section. For each iteration of the PSE, we ranked the FC and FCD fits for each value of *G* and chose the optimal *G* value based on the best combined rank. Then we chose the value of *G* that most frequently (mode) produced the best combined FC/FCD fit. We also used the regional *J_i_* values for the best fitting value of *G*, found by the FIC algorithm. We refer to global coupling and regional inhibitory connection weights (97 variables total) as the “TVB parameters” collectively. Note that global coupling was used to maximize fit with empirical fMRI, while the fitting target for regional inhibitory connection strength was the average firing rate of the excitatory population at each region.

### Partial least squares

Partial least squares (PLS) is a multivariate statistical method that relates two sets of variables by identifying linear combinations of variables in both sets that maximally covary together (McIntosh and Lobaugh, 2004; McIntosh and Mišić, 2013). We used PLS to find optimal relationships between a set of brain variables and either a study design (mean-centering PLS) or a set of behavioral variables (behavioral PLS). In PLS, singular value decomposition is used to find orthogonal latent variables that explain the maximal amount of covariance between brain variables and design or behavior variables. For each latent variable, brain saliences are calculated for each brain region that indicate the degree to which each region contributes to the relationship between brain and design/behavior expressed by the latent variable. In mean-centering PLS, design saliences indicate the group, condition, or group × condition profiles that best describe the relationship between the set of brain and design variables. In behavioral PLS, behavior saliences indicate the profile of behavior variables that best characterize the relationship between brain and behavior variables. Brain scores are calculated for each subject and latent variable by multiplying the matrix of brain variables by the brain saliences. The brain score indicates the degree to which each participant contributes to each latent variable. Last, singular values are the covariance between brain and behavior/grouping variables. They can also be evaluated as a percentage of the total covariance between measures that each latent variable accounts for.

In our study, we used mean centering PLS to determine the relationship between several brain measures and group status. The brain measures considered were a vectorized version of the upper triangle of the SC/FC matrices, FA values from all voxels within our white matter mask, and TVB parameters (global coupling and regional inhibitory connection strength values). For each brain measure, an omnibus PLS was performed that found the optimal contrasts in brain measures between group membership. We also used behavioral PLS to determine the relationships between the TVB parameters (global coupling and regional inhibitory connection strength) and factor scores from a set of patient outcome variables.

Permutation testing was used to determine the significance of each latent variable. Rows of the data matrix were randomly reordered, and the singular value was recalculated. This was done 1000 times, creating a distribution of singular values. Then a *p*-value for the original singular value was calculated by taking the proportion of singular values from the sampling distribution that were larger than the original singular value. The *p*-value can be thought of as the probability of obtaining a singular value of this size under the null hypothesis that there is no association between brain measure and design/behavior.

Bootstrapping was used to estimate the reliability of each brain salience. Participants were randomly resampled 1000 times with replacement, while respecting group membership. The resampled matrices were used to recalculate the singular vector decomposition, producing a sampling distribution for the weights in the singular vectors. The SE was calculated from this sampling distribution, reflecting the stability of the weight regardless of which participants are included in the analysis. Then a bootstrap ratio (BSR) was calculated for each brain salience (voxel, brain region, connection) by dividing the brain salience by its bootstrap-estimate SE. BSR is akin to *z* score (2.0 corresponds to approximately a 95% confidence interval) but is interpreted in terms of the reliability of the parameter rather than null hypothesis testing. Confidence intervals were calculated around design/behavior salience using the percentiles derived from the sampling distribution.

## Results

### Outcome measures

Our PCA of background and outcome variables identified the following two factors: (1) TBI Symptoms and (2) Age and Cognition (Table 5), consistent with a previously reported PCA of neuropsychological outcome variables in mTBI patients (Levin et al., 2013). The CT/MRI subgroups did not significantly differ on the TBI Symptoms factor (*t*_(34)_ = 0.73, *p *=* *0.47) or the Age and Cognition factor (*t*_(34)_ = 0.30, *p *=* *0.76). We also tested for group differences on the individual variables contributing to both factors. We found that the CT/MRI-positive patients were significantly older than the CT/MRI-negative patients (*t*_(42)_ = −2.5, *p *<* *0.01) and comparison participants (*t*_(48)_ = 4.3, *p* ≤ 0.01). The CT/MRI-negative patients were also significantly older than the comparison participants (*t*_(64)_ = 2.2, *p *=* *0.03). A χ^2^ test of independence indicated that the CT/MRI-positive patients experienced a greater loss of consciousness than the CT/MRI-negative patients (χ(1)^2^ = 8.6, *p* = 0.01). There were no other significant differences between groups (*p *>* *0.05; Table 1).

### Empirical brain differences between patient subgroups and comparison participants

#### Structural connectivity

**Figure 2.**
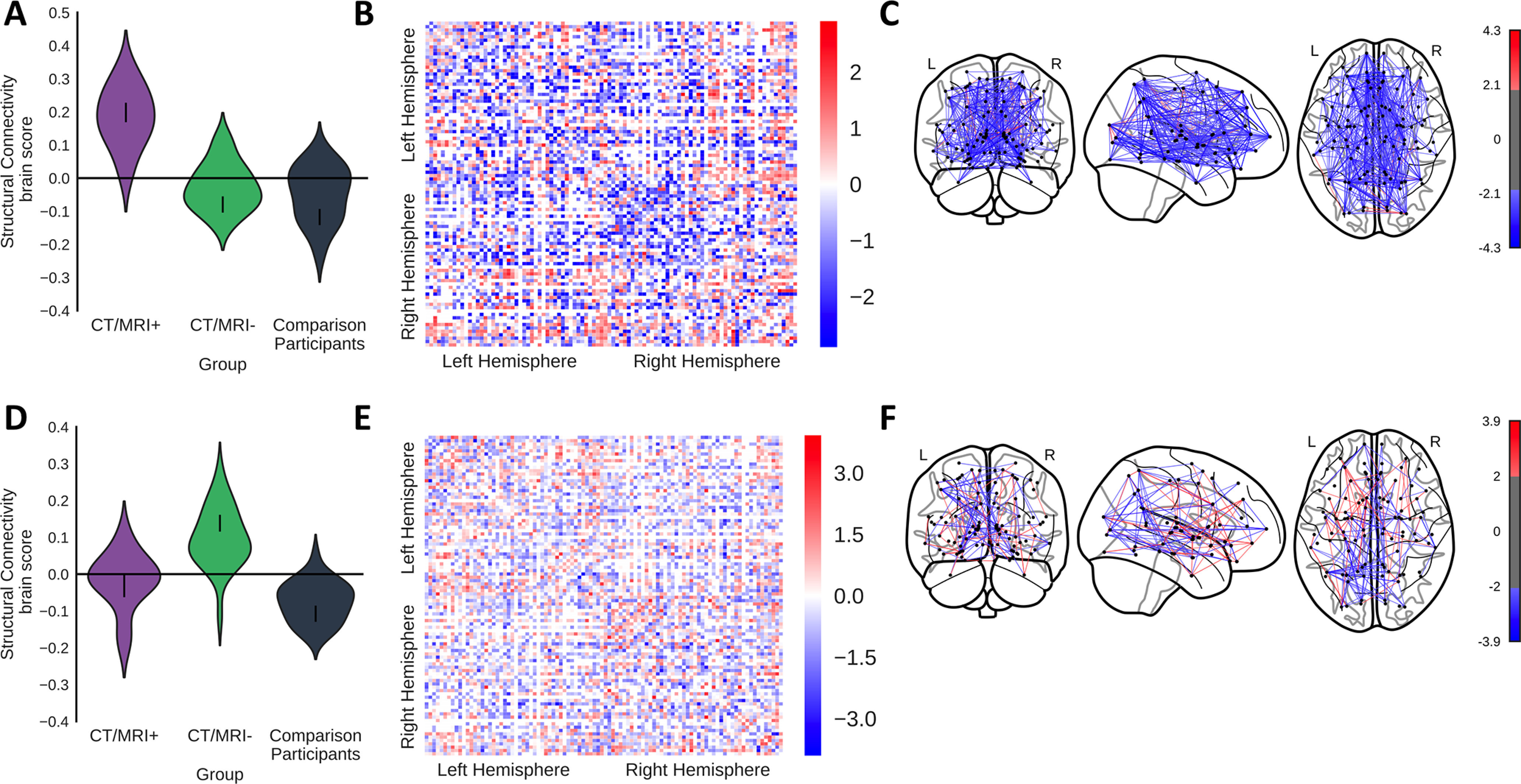
A group-comparison PLS distinguished the SC of CT/MRI-positive and CT/MRI-negative patients from comparison participants. ***A–F***, The first latent variable (*p *<* *0.0001, 67% covariance, singular value = 0.19) shows differentiation of CT/MRI-positive patients from the comparison participants (***A–C***), while the second latent variable (*p *=* *0.016, 33% covariance, singular value = 0.13) differentiated CT/MRI-negative patients from the comparison participants (***D–F***). ***A***, ***D***, Violin plots show the distribution of brain scores for each group. Brain scores indicate the degree to which participants express the pattern of SC shown in ***B*** and ***E***. Error bars are bootstrap-estimated 95% confidence intervals. ***B***, ***E***, Bootstrap ratios, which are a linear combination of SC weighted by how strongly they contribute to the latent variable are shown. Bootstrap ratios may be interpreted similar to *z* scores (>2.0, akin to *p *<* *0.05), so regions with bars exceeding the dashed line may be considered to reliably contribute to the latent variable. ***C***, ***F***, Regional inhibitory connection strength bootstrap ratios that reliably contribute to the latent variable (>2) from ***B*** and ***E*** projected onto a brain.

A group comparison PLS assessed potential differences in SC weights between the CT/MRI patient subgroups and comparison participants. The first latent variable (*p *<* *0.0001, 67% covariance, singular value = 0.19) distinguished the CT/MRI-positive patients from comparison participants (Fig. 2*A–C*), indicating that CT/MRI-positive patients had lower SC weights relative to the comparison participants in many voxels, indicating a global effect. The second latent variable (*p *=* *0.016, 33% covariance, singular value = 0.13) distinguished CT/MRI-negative patients from comparison participants (Fig. 2*D–F*) showing that the CT/MRI-negative patients also had primarily lower SC than comparison participants especially in connections involving occipital regions. These analyses were repeated with age regressed from the SC values, and the results did not change appreciably.

#### Functional connectivity and functional connectivity dynamics

A group comparison PLS comparing functional connectivity across the CT/MRI patient subgroups and comparison participants did not find any significant differences (*p *=* *0.58, 61.7% covariance, singular value = 3.1). A one-way ANOVA did not discriminate the patient groups or comparison subjects on the variance of their FCD matrices (*F*_(2,77)_ = 0.1, *p *=* *0.91). These analyses were repeated with age regressed from the FC values, and the results did not change appreciably.

#### Fractional anisotropy

**Figure 3.**
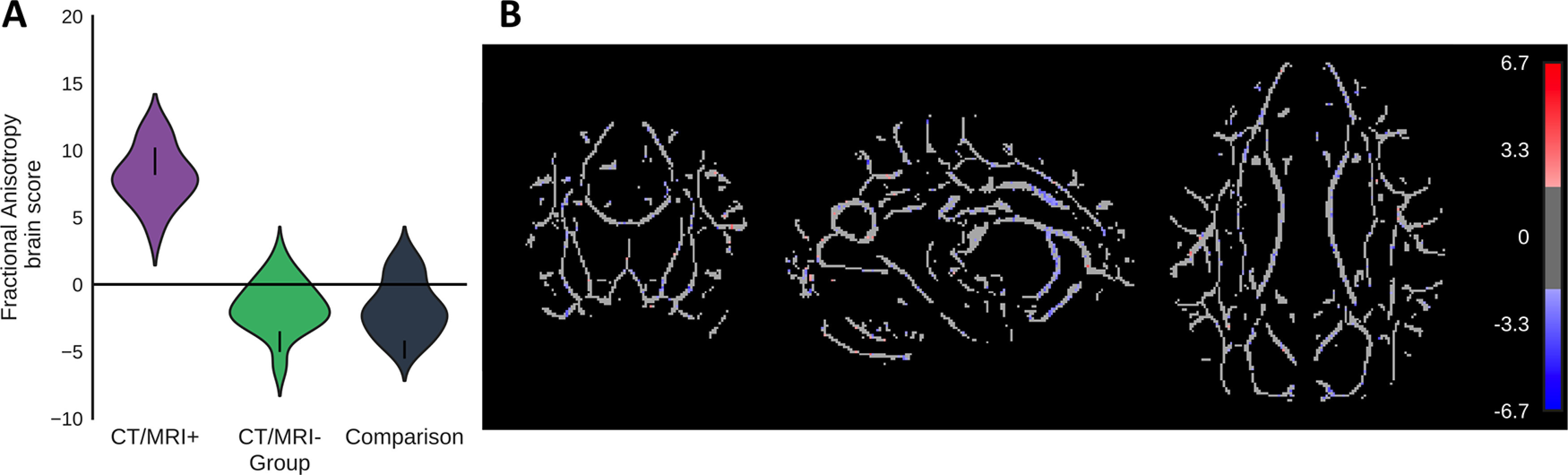
A group-comparison PLS distinguished the CT/MRI-positive and CT/MRI-negative patients from comparison participants via their fractional anisotropy. ***A***, ***B***, The first latent variable (*p *=* *0.01, 65% covariance, singular value = 8.2) that distinguished CT/MRI-positive patients from the comparison participants. ***A***, Violin plot shows the distribution of brain scores for each group. Brain scores indicate the degree to which participants express the pattern fractional anisotropy shown in ***B***. Error bars are bootstrap-estimated 95% confidence intervals. ***B***, Bootstrap ratios, which are a linear combination of voxelwise fractional anisotropy weighted by how strongly they contribute to the latent variable. Bootstrap ratios are superimposed onto a white matter skeleton. Bootstrap ratios may be interpreted similar to *z* scores (>2.0, akin to *p *<* *0.05), so only voxels with bootstrap ratios >2 are illustrated.

A group comparison PLS compared fractional anisotropy across the whole-brain white matter skeleton (Fig. 3). The first latent variable (*p *=* *0.01, 65.1% covariance, singular value = 8.2) showed that CT/MRI-positive patients had lower FA values than the comparison group, especially in the left cingulum and anterior corona radiata. The second latent variable indicated that CT/MRI-negative patients had primarily lower FA values than comparison participants, though they did not reach significance (*p *=* *0.09, 34.9% covariance, singular value = 6.0). These analyses were repeated with age regressed from the FA values, and the results did not change meaningfully.

### TVB model fitting

All patients and comparison participants showed good fits between empirical and simulated resting-state fMRI, as assessed by high uncentered Pearson’s correlation of FC and low Kolmogorov–Smirnov distance between FCD matrices. The simulated FC and FCD matrices of all participants were inspected visually to ensure that they appeared as reasonable FC/FCD matrices qualitatively. We also screened for outliers by converting the FC and FCD fits to the *z* score, finding no participants with a z-scored FC fit lower than −3 (minimum = −2.4) and no participants with a *z*-scored FCD fit >3 (maximum = 2.6). There were no significant differences among the three groups on the fitting metrics (Table 5).

**Figure 4.**
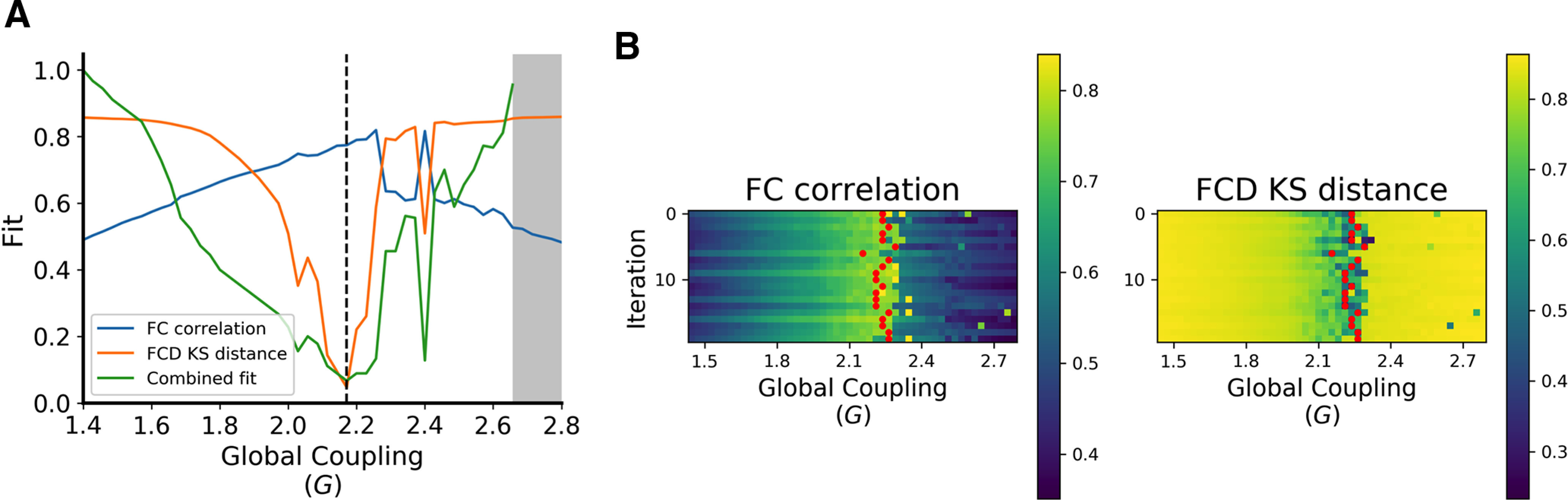
Summary of modeling fitting procedure. ***A***, The parameter space exploration map for a representative subject given a single iteration. The gray area denotes values of global coupling for which the model fails to converge because it becomes hyperexcited. The black dashed line represents the optimal value of global coupling. Note that combined fit (green) is defined by the sum of the FC and FCD fits ranked across all other values of global coupling at which the model converged. ***B***, The full-parameter space results for the same representative subject. Each grid shows model fits across all values of *G* on the *x*-axis, and iterations with randomized initial conditions on the *y*-axis. On the left, fits are defined by the uncentered correlation of the upper triangle of the empirical and simulated FC matrices. On the right, Kolmogorov–Smirnov (KS) distance between the upper triangles of the empirical and simulated FCD matrices defines fits. The red dots represent the optimal fit for each iteration.

The parameter search space showed steady improvements in fit (increased FC correlation and decreased FCD Kolmogorov–Smirnov distance) as global coupling was increased until a best-fitting global coupling value was reached (Fig. 4). After this point, the model becomes multistable and eventually the FIC tuning algorithm is no longer able to maintain biologically realistic firing rates of ∼3 Hz. Across multiple iterations, our model showed acceptable consistency in choosing the optimal value of global coupling across iterations (Fig. 4, Table 6).

**Table 6:** Model-fitting results

Fitting metric	Descriptive statistic	CT/MRI-positive (*n* = 14)	CT/MRI negative (*n* = 30)	Comparison (*n* = 36)	Significance
Functional connectivity, unlefted correlation	Mean	0.66	0.66	0.68	*F*_(2,77)_ = 0.32, *p* = 0.73
	SD	0.11	0.09	0.12	
	Minimum	0.50	0.33	0.35	
	Maximum	0.79	0.84	0.88	
Functional connectivity dynamics, Kolmogorov–Smirnov distance	Mean	0.13	0.12	0.12	*F*_(2,77)_ = 0.03, *p* = 0.97
	SD	0.07	0.14	0.13	
	Minimum	0.04	0.03	0.03	
	Maximum	0.31	0.70	0.74	
Iterations optimal solution was chosen for	Mean	37.0%	34.5%	34.3%	*F*_(2,77)_ = 0.15, *p* = 0.86
	SD	13.7%	16.2%	14.9%	
	Minimum	15.0%	15.0%	15.0%	
	Maximum	75.0%	90.0%	90.0%	

### TVB model group differences

#### Combined TBI patients versus comparison subjects

**Figure 5.**
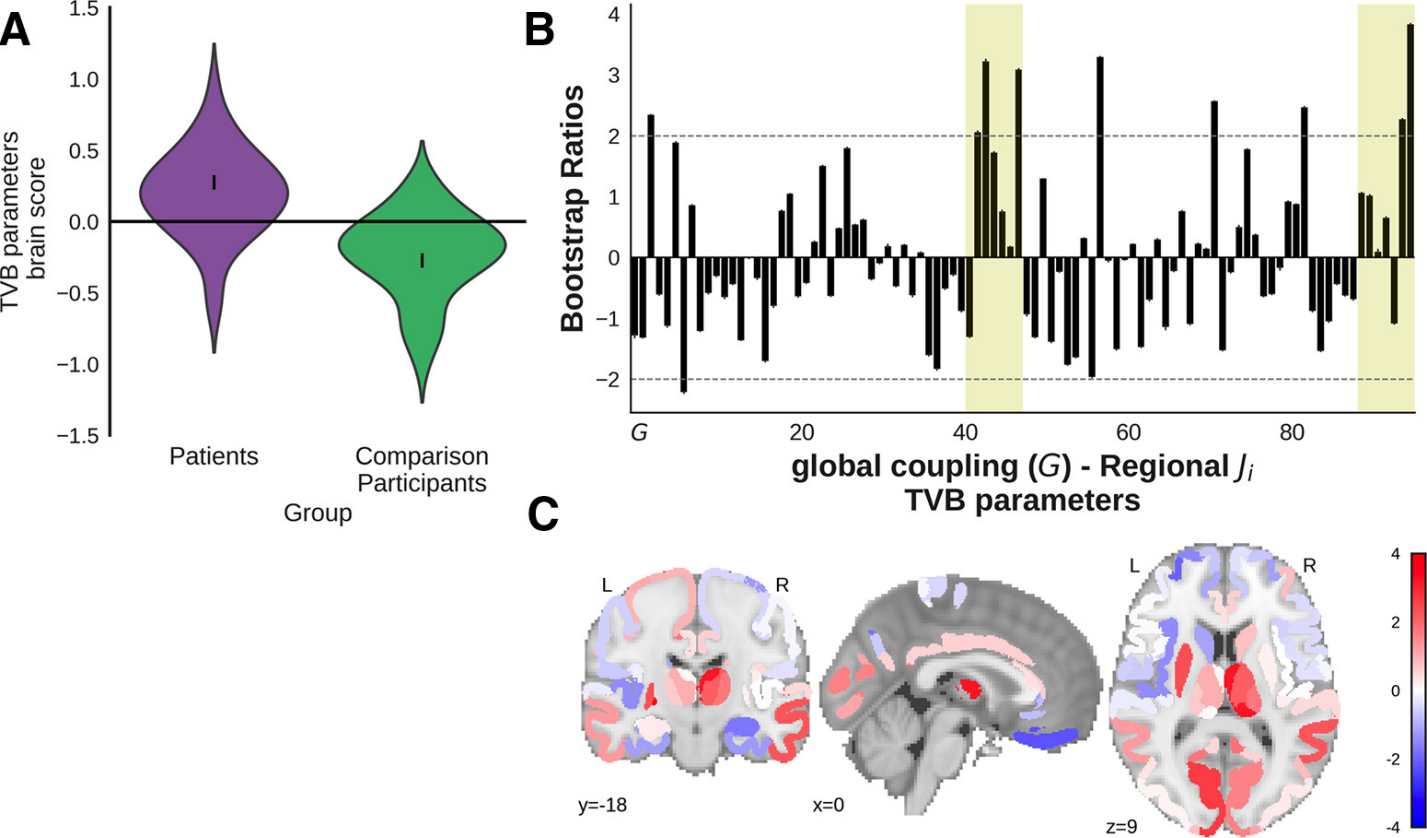
Group comparison PLS of TVB parameters (*G* and regional inhibitory connection strengths) across patients (combined CT/MRI-positive and CT/MRI-negative subgroups) and comparison participants (*p *=* *0.026, singular value = 0.30). Patients showed mostly higher inhibitory connection strength relative to comparison participants, particularly in the subcortical regions. ***A***, The violin plot shows the distribution of brain scores for each group. Brain scores indicate the degree to which participants express the pattern of global coupling and regional local inhibitory connection strength shown in ***B***. Error bars are bootstrap-estimated 95% confidence intervals. ***B***, Bootstrap ratios, which are a linear combination of global coupling and regional local inhibitory connection strength weighted by how strongly they contribute to the latent variable. Bootstrap ratios may be interpreted similar to *z* scores (>2.0, akin to *p *<* *0.05), so regions with bars exceeding the dashed line may be considered to reliably contribute to the latent variable. Error bars are 1 SE. Bars representing subcortical regions are shaded. ***C***, Regional inhibitory connection strength bootstrap ratios from ***B*** projected onto a glass brain.

A group comparison PLS compared the TVB parameters (global coupling and regional inhibitory connection strengths) of the comparison participants (*n* = 36) and the combined CT/MRI-positive and CT/MRI-negative patient groups (*n* = 44). A significant latent variable (*p *=* *0.026, singular value = 0.30; Fig. 5) distinguished the groups, showing that the TBI patients had primarily higher inhibitory connection strengths, especially in bilateral subcortical regions. Regions that were reliably contributing (bootstrap ratio, >2) to the relationship included the bilateral pallidum, a right thalamic ROI with major temporal connections and right thalamic ROI with major frontal connections, right superior temporal cortex, left inferior cortex, and left putamen. The results were not significantly affected when age was regressed from the variables and the analysis repeated.

#### CT/MRI-positive and CT/MRI-negative patients versus comparison participants

**Figure 6.**
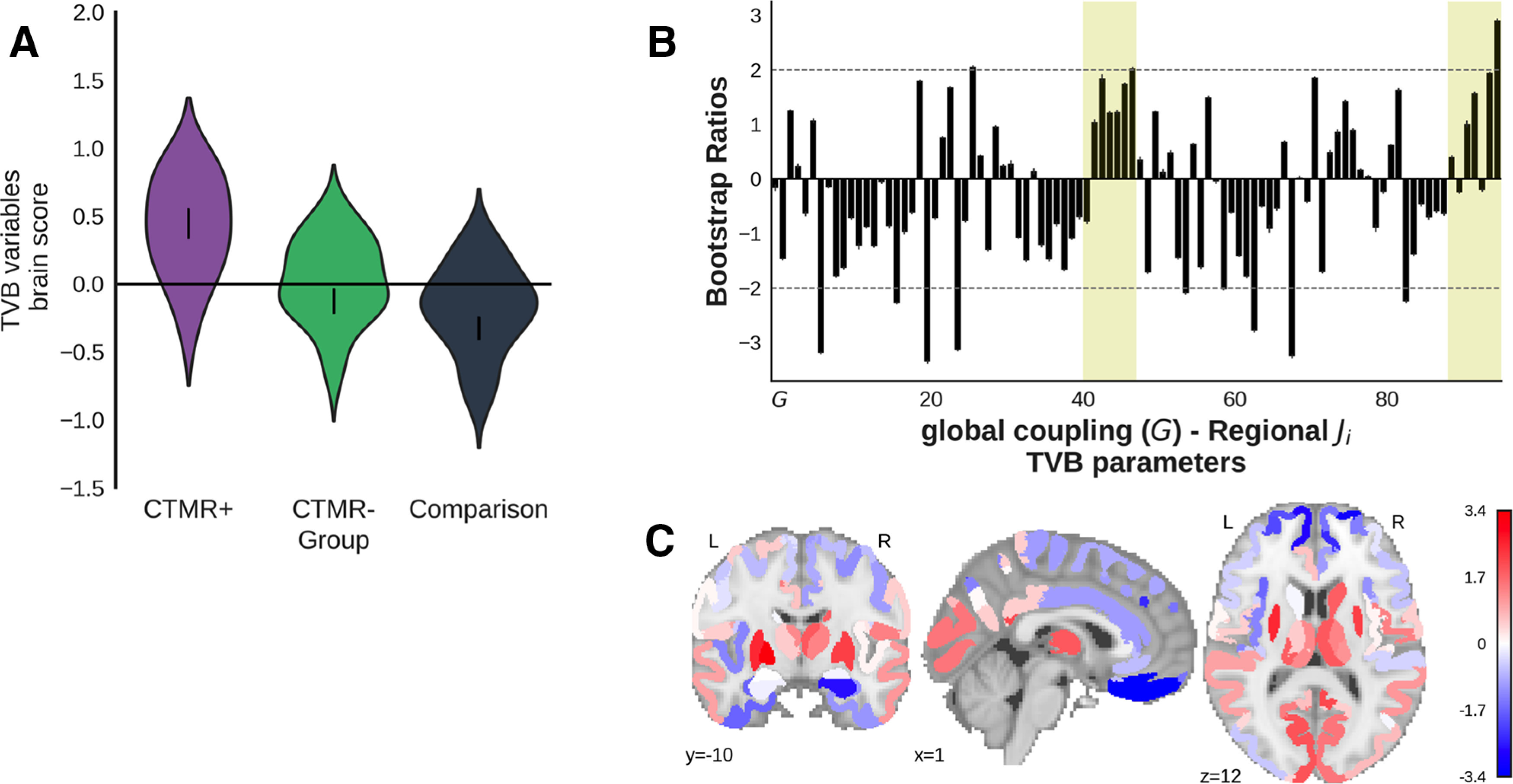
Group-comparison PLS of TVB parameters (*G* and regional inhibitory connection strengths) across CT/MRI-positive patients, CT/MRI-negative patients, and comparison participants. ***A–C***, The first latent variable (*p *=* *0.03, 70.9% covariance, singular value = 0.43) that differentiated CT/MRI-positive patients from comparison participants. ***A***, Violin plot shows the distribution of brain scores for each group. Brain scores indicate the degree to which participants express the pattern of global coupling and regional local inhibitory connection strength shown in ***B***. Error bars are bootstrap-estimated 95% confidence intervals. ***B***, Bootstrap ratios, which are a linear combination of global coupling and regional local inhibitory connection strength weighted by how strongly they contribute to the latent variable. Bootstrap ratios may be interpreted similar to *z* scores (>2.0, akin to *p *<* *0.05), so regions with bars exceeding the dashed line may be considered to reliably contribute to the latent variable. Error bars are 1 SE. Bars representing subcortical regions are shaded. ***C***, Regional inhibitory connection strength bootstrap ratios from ***B*** projected onto a brain.

A group comparison PLS compared the TVB parameters (global coupling and regional inhibitory connection strengths) of the CT/MRI-positive patients (*n* = 14), CT/MRI-negative patients (*n* = 30), and comparison participants (*n* = 36). The first significant latent variable (*p *=* *0.03, 70.9% covariance, singular value = 0.43; Fig. 6*A–C*) distinguished the CT/MRI-positive patients from comparison participants, showing that they had primarily lower cortical and higher subcortical inhibitory connection strengths relative to the comparison subjects. Regions reliably lower in CT/MRI-positive patients compared with comparison participants (bootstrap ratio, less than −2) included the left dorsomedial prefrontal cortex, left medial prefrontal cortex, left prefrontal polar cortex, left anterior insula, right centrolateral prefrontal cortex, right medial prefrontal cortex, and right orbitoinferior prefrontal cortex. On the other hand, the left/right pallidum were reliably higher in CT/MRI-positive patients relative to comparison participants. The dot product of the brain salience vector from the first latent variable (Fig. 6) and the brain salience vector from the group comparison PLS comparing the combined patients from comparison subjects (Fig. 5) was high (*r* = 0.77), indicating that the CT/MRI-positive patients were likely largely responsible for driving the group difference. These analyses were repeated with age regressed from the TVB parameters, and the results did not change meaningfully.

### TVB–behavior relationships

**Figure 7.**
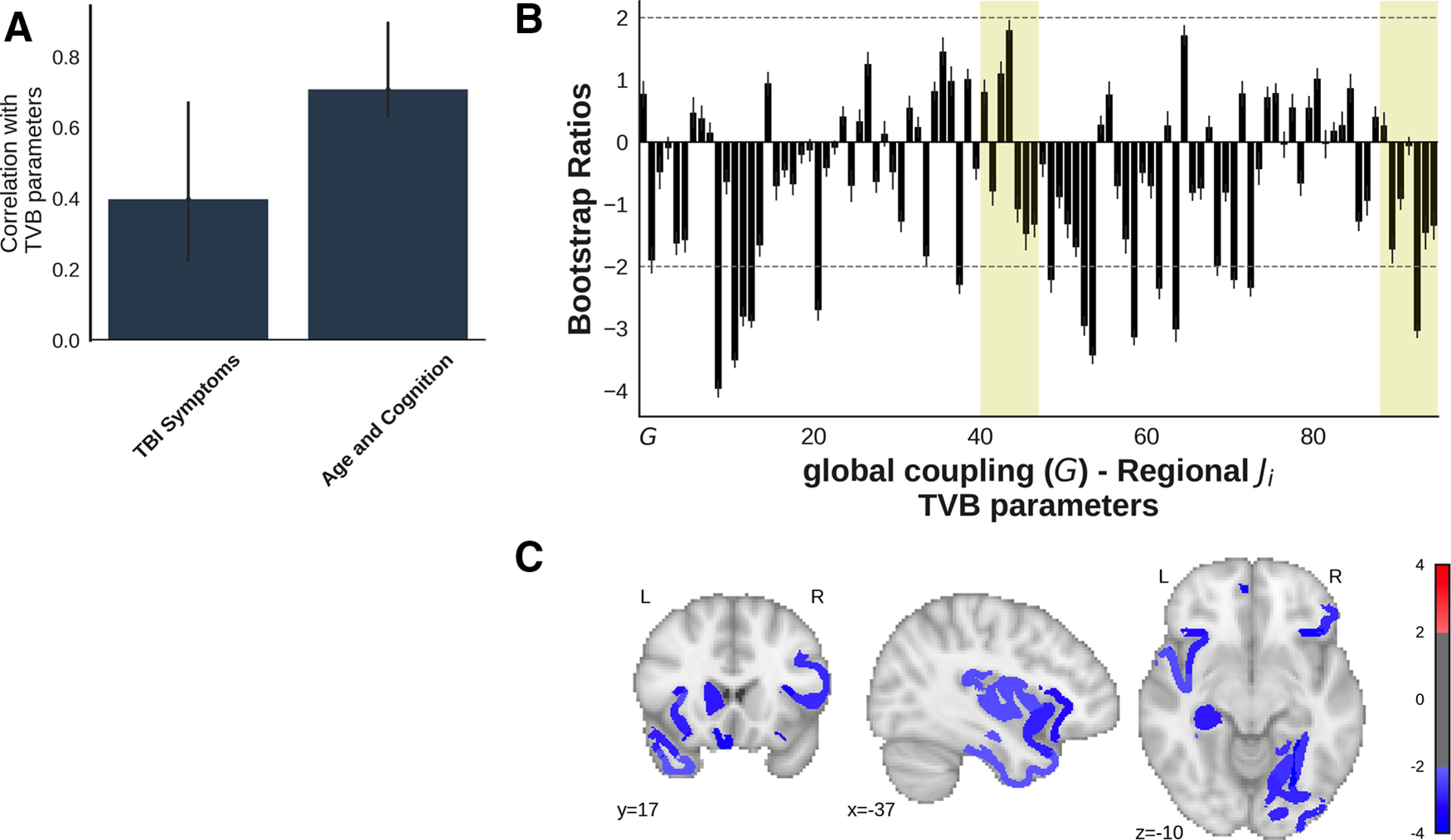
A behavioral PLS analysis assessed the associations between the TBI Symptoms and Age and Cognition factors and TVB parameters (global coupling and regional inhibitory connection strengths) in the patients (combined CT/MRI-positive and CT/MRI-negative subgroups). The first significant variable is illustrated (*p *=* *0.02, 74% covariance, singular value = 2.4) ***A***, The bars represent the correlation between each factor and the pattern of TVB parameters shown in the corresponding bar graph in ***B***. The error bars represent 95% confidence intervals, so the error bars of variables significantly contributing to the latent variable do not cross zero. ***B***, Bootstrap ratios, which are a linear combination of global coupling and regional local inhibitory connection strength weighted by how strongly they contribute to the latent variable. Bootstrap ratios may be interpreted similar to *z* scores (>2.0, akin to *p *<* *0.05), so regions with bars exceeding the dashed line may be considered to reliably contribute to the latent variable. Error bars are 1 SE. Bars representing subcortical regions are shaded. ***C***, Regional inhibitory connection strength bootstrap ratios from ***B*** that reliably contribute to the latent variable (>2) projected onto a brain.

A behavioral PLS analysis compared the correlations between the TVB parameters and the TBI Symptoms and Age and Cognition factors in all of the patients (*N* = 36). The analysis showed higher scores on the TBI Symptoms (indicating more symptoms) and Age and Cognition (indicating older age and poorer cognitive performance) factors were related to primarily lower regional inhibitory connection strength (*p *=* *0.02, 74% covariance, singular value = 2.4; Fig. 7). Regions reliably contributing to the latent variable (bootstrap ratios, less than −2) included the left/right gustatory cortex, the left caudate nucleus, secondary auditory cortex, primary visual cortex, hippocampus, orbitomedial prefrontal cortex, anterior insula, and temporal polar cortex, as well as the right frontal eye field, ventral temporal cortex, anterior visual area (ventral part), ventrolateral premotor cortex, gustatory cortex, and inferior temporal cortex.

**Figure 8.**
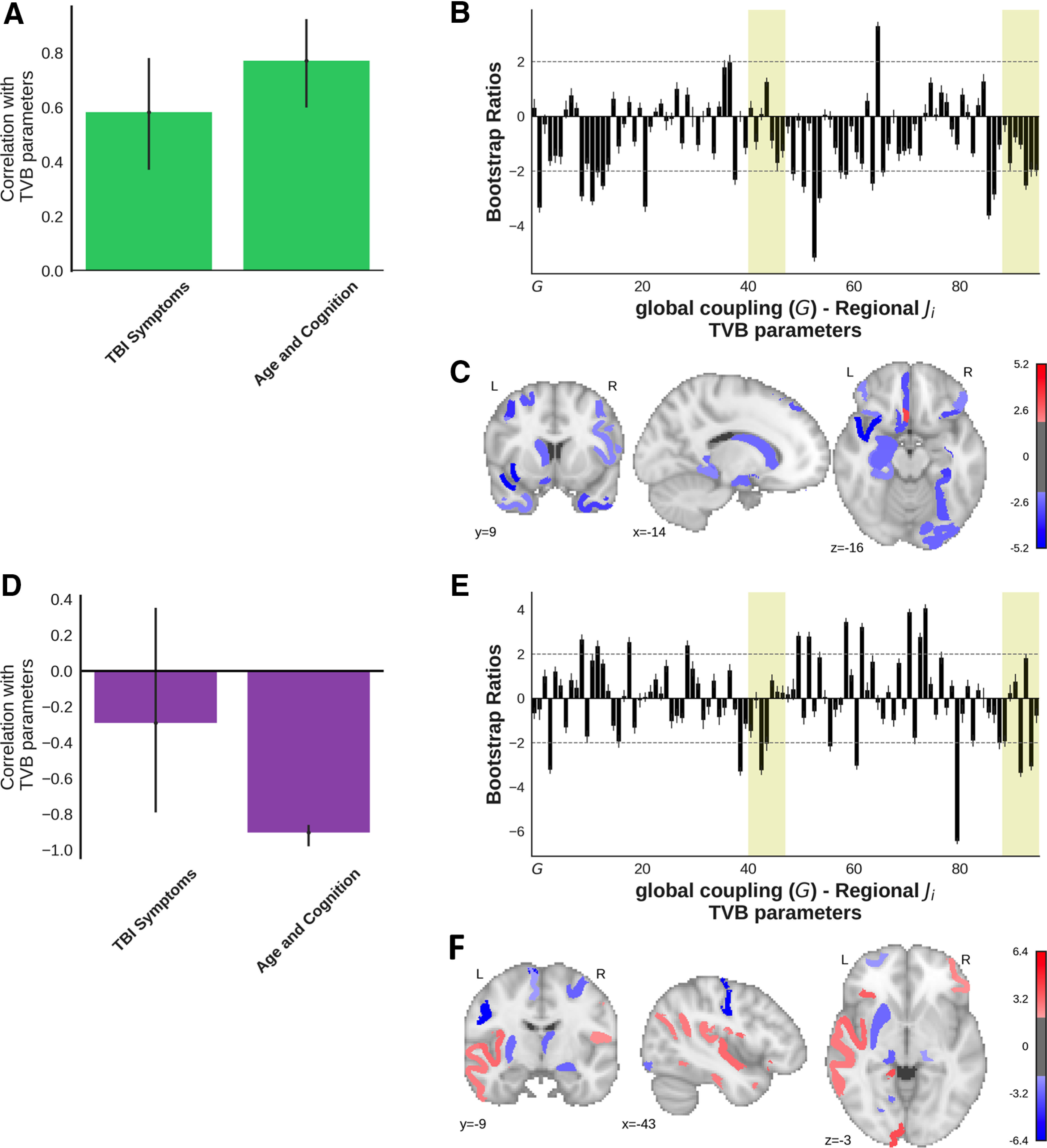
***A–F***, Within-group behavioral PLS analyses show the relationships between the TBI Symptoms and Age and Cognition factor scores and the TVB parameters (*G* and regional inhibitory connection strengths) for the CT/MRI-negative (***A–C***) and CT/MRI-positive patients (***D–F***). ***A–C*** show the first latent variable (*p *=* *0.005, 73.1% covariance, singular value = 3.0) for the within CT/MRI-negative patients, while ***D–F*** illustrate the first latent variable (*p *=* *0.11, 67.6% covariance, singular value = 3.7) for the CT/MRI-positive patients. ***A***, ***D***, The bars represent the correlation between each factor with the pattern of TVB parameters shown in the corresponding bar graph ***B***. The error bars represent 95% confidence intervals, so the error bars of variables significantly contributing to the latent variable do not cross zero. ***B*** and ***E*** show bootstrap ratios, which are a linear combination of global coupling and regional local inhibitory connection strength weighted by how strongly they contribute to the latent variable. Bootstrap ratios may be interpreted similar to *z* scores (>2.0, akin to *p *<* *0.05), so regions with bars exceeding the dashed line may be considered to reliably contribute to the latent variable. Error bars are 1 SE. Bars representing subcortical regions are shaded. ***C***, ***F***, Regional inhibitory connection strength bootstrap ratios that reliably contribute to the latent variable (>2) from ***B*** and ***E*** projected onto a brain.

Within-group PLS models were calculated to determine the contributions of the CT/MRI-positive and CT/MRI-negative groups to the omnibus test shown in Figure 8. Both PLS analyses compared TBI Symptoms and Age and Cognition factor scores to the TVB parameters (global coupling and regional inhibitory connection strength) using either the CT/MRI-positive or CT/MRI-negative patients. The within-CT/MRI-negative behavioral PLS produced a single significant latent variable (*n* = 25; *p *=* *0.005, 73.1% covariance, singular value = 3.0; Fig. 8*A–C*), indicating that higher TBI Symptoms and Age and Cognition scores were related to lower inhibitory connection strength. Regions reliably contributing to the latent variable (bootstrap ratios, less than −2) included the right temporal polar cortex, orbitomedial prefrontal cortex, parahippocampal cortex, anterior visual area, ventral temporal cortex, and frontal eye field, as well as the left anterior insula, orbitomedial prefrontal cortex, hippocampus, frontal eye field, dorsolateral prefrontal cortex, and caudate nucleus. The left subgenual cingulate gyrus showed the inverse association with the factor scores compared with the other regions (bootstrap ratio, >2). The same analysis within the CT/MRI-positive group did not produce a significant latent variable (*n* = 11, *p *=* *0.11, 67.6% covariance, singular value = 3.7; Fig. 8*D–F*). We calculated the dot product of the brain salience vectors from the combined group PLS model (Fig. 7) and the two within-group analyses (Fig. 8) to determine which subgroup was responsible for driving the effect observed when groups were combined. The dot product between the combined model and the CT/MRI-negative model was very high (*r* = 0.90). The same dot product was significantly lower (*r* = −0.43) between the combined model and the CT/MRI-positive group (*z *=* *4.68, *p *<* *0.0001), suggesting that the CT/MRI-negative group was primarily responsible for the observed associations between TBI Symptoms and Age and Cognition factor scores and regional inhibitory connection strengths.

## Discussion

### Overview

Concussion outcomes are variable and difficult to predict. Few studies have looked longitudinally at patients with well characterized acute-phase and chronic-phase assessments, and fewer still have combined multimodal imaging with a computational approach. Here we combined the strengths of a well characterized sample with multimodal imaging and computational approaches to reveal relationships between local neural dynamics and chronic patient outcomes. We found that the CT/MRI-positive TBI patients showed lower cortical, but higher subcortical, inhibitory connection strengths relative to the comparison participants. We did not distinguish CT/MRI-negative patients from the comparison participants based on their TVB parameters; however, lower regional inhibitory connection strengths were related to higher scores on the TBI Symptoms and Age and Cognition factors assessed 6 months postinjury in these patients. Together, our results show proof of concept that local brain dynamics modeled in TVB are sensitive to semiacute TBI pathology and predictive of chronic outcomes.

### Group comparisons

The CT/MRI-positive patients displayed higher subcortical, but lower cortical, inhibitory connection strengths relative to the comparison participants. We speculate that lower inhibitory connection strength may reflect excitotoxicity or inhibitory dysregulation, which are known to play a role in secondary injury after TBI (Yi and Hazell, 2006; Van Horn et al., 2017). Indeed, a study of tumor patients (Aerts et al., 2018) that used a model similar to our own demonstrated reduced inhibitory connection strength in tumor regions relative to comparison participants, which they posited to reflect the role of excitotoxicity in the pathogenesis of glaucoma (Casson, 2006). Aerts et al. (2018) also observed increased inhibitory connection strengths in the nontumor regions of their patients compared with controls, paralleling the increased inhibitory connection strengths we observed in the subcortical regions of the CT/MRI-positive patients compared with comparison participants. Higher subcortical inhibitory connection strengths may reflect a distal effect of small cortical lesions or serve a protective function, as CT/MRI-positive patients did not score more highly than CT/MRI-negative patients on the TBI Symptoms factor. Future studies using neuroimaging with finer spatial resolution could quantitatively assess the sensitivity of the regional inhibitory connection strength parameter of our model to the cellular processes underlying changes to excitation/inhibition caused by TBI.

The CT/MRI-negative patients were not significantly differentiated from the comparison participants by their TVB parameters. This was unexpected as a study of mTBI patients experiencing active PCS symptoms (who were screened for focal lesions) found decreased inhibitory connection strengths relative to comparison participants (Good T, McIntosh AR, Levine B, unpublished observations). Notably, the present work investigates TBI patients scanned 1–2 weeks postinjury, while the other study considers patients in the chronic phase postinjury, which may contribute to the discrepant findings. It is also possible that other factors obfuscated a potential group difference between CT/MRI-negative patients and comparison participants. For example, a history of substance abuse and other neuropsychiatric disorders, such as depression, were not exclusion criteria in the present study despite exerting effects via excitotoxicity (Miller et al., 2009; Walker and Dantzer, 2014), similar to mTBI. While this feature of our data increases the generalizability of our findings, it may also have limited our sensitivity to pathophysiology caused by TBI.

Our empirical SC and FA findings suggested the CT/MRI-positive and CT/MRI-negative patients were characterized by more disconnected structural connectomes than the comparison participants. These results are in line with a previous study of the white matter integrity of an overlapping patient sample (Yuh et al., 2014). In addition, previous studies of humans (Imms et al., 2019; Kuceyeski et al., 2019) and animals (Meningher et al., 2020) have found that the SCs of TBI patients are more highly segregated than those of control subjects. Modeling studies suggest that structural disconnection in TBI patients produces reduced metastability, which is linked to excitation–inhibition imbalance (Hellyer et al., 2015). Similarly, the CT/MRI-positive patients demonstrated structural disconnection as well as altered local inhibitory dynamics compared with comparison participants.

When interpreting our findings, it should be considered that approximately one-third of our TBI sample (14 of 44 patients) showed signs of acute traumatic intracranial lesions on CT or MRI scans on presentation in the emergency department. This classification of CT/MRI-positive versus CT/MRI-negative may be contrasted with previous work considering “complicated” mTBI solely on the basis of a positive acute-phase CT scan (Iverson et al., 2012; Voormolen et al., 2019). Many of our CT/MRI-positive patients were classified on the basis of very subtle MRI lesions at 3 T, such as one or two subtle isolated foci of hemorrhagic axonal injury. These patients would likely be classified as uncomplicated if we had used CT alone. In fact, this nuance is supported by early work that found dwMRI evidence of white matter damage in patients classified as uncomplicated by CT scan alone (Arfanakis et al., 2002; Bazarian et al., 2007; Chu et al., 2010).

### TVB–behavior relationships

Lower inhibitory connection strengths were associated with higher scores on the TBI Symptoms and Age and Cognition factors in the CT/MRI-negative group. The effect included many regions, including cortical and subcortical structures, and indicated that lower inhibitory connection strengths were related to more severe TBI outcomes, older age, less education, and lower cognitive performance. This effect is aligned with other large-scale modeling work that showed structural disconnection associated with decreased metastability, indicating excitation–inhibition imbalance, to be related to poorer cognitive performance in TBI patients (Hellyer et al., 2015). Empirical studies using MRS have also found that elevated concentrations of the neuromodulatory factor glutamate during the semiacute phase were predictive of poorer chronic outcomes (Shutter et al., 2004; Eisele et al., 2020).

In the CT/MRI-positive patients we did not observe a correlation between inhibitory connection strengths and the TBI Symptoms factor as expected. We suspect that this was because of a lack of statistical power (*n* = 11) in the within-group behavioral PLS (Fig. 8*D–F*). Forthcoming large-scale longitudinal and multisite data acquisitions [e.g., full Track-TBI LONG (https://tracktbi.ucsf.edu/) sample] will be better equipped to establish robust correlations between parameters from brain network models and chronic TBI outcome.

### Limitations and conclusions

We note that our study is limited by its modest sample size and the lack of longitudinal imaging data to track recovery. Our exploratory findings will need to be confirmed in larger independent samples. Specifically, future studies should test whether a lower inhibitory connection strength is indeed predictive of the development of persistent postconcussion symptoms. Additionally, we acknowledge the lack of racial diversity (79% white) and the gender imbalance (65% male) of our patients. Our sample, obtained by an emergency department convenience sample, will limit the generalizability of our findings to more mild concussions that do not require hospitalization. Our choice of a relatively coarse (96 ROIs) parcellation scheme may have also limited sensitivity. Future work should explore the effect of multiple parcellation schemes on modeling results. Similarly, future work with larger sample sizes may allow for subtyping moderate to severe TBI patients by lesion type and location, which would allow for greater power in detecting changes to local network dynamics. Regarding our network model, we acknowledge that a mechanistic interpretation may not be possible as multiple processes at the micro level may contribute to similar observations at the macro level. For example, local excitatory connection strengths may produce dynamics similar as those observed through variations to recurrent inhibitory connection strengths. We did not vary local excitatory connection strengths in our parameter space exploration to ensure that the model remained identifiable.

In conclusion, we used large-scale brain modeling to detect differences in local inhibitory connection strengths among CT/MRI-positive TBI patients in the semiacute phase and comparison participants. We did not distinguish semiacute CT/MRI-negative patients from the comparison participants based on their inhibitory connection strengths; however, lower inhibitory connection strengths were associated with more severe clinical outcomes, older age, and poorer cognitive performance at a 6 month follow-up in these patients. The result suggests large-scale connectome-based models may be sensitive to pathophysiological changes in semi-acute phase TBI patients and predictive of their chronic outcomes.

## Synthesis

Reviewing Editor: Viji Santhakumar, University of California Riverside Decisions are customarily a result of the Reviewing Editor and the peer reviewers coming together and discussing their recommendations until a consensus is reached. When revisions are invited, a fact-based synthesis statement explaining their decision and outlining what is needed to prepare a revision will be listed below. The following reviewer(s) agreed to reveal their identity: NONE.

The study examines how computational modeling of individualized brains using imaging data from patients can be used to relate differences in modeling parameters and dynamics to variable outcomes after brain injury. Overall, the reviewers are positive about the approach and the ability of the simulations to generate mechanistic predictions regrading connectivity. While this is a potentially useful approach, there are specific issues that need additional acknowledgement and /or clarification including the limitations associated with drawing conclusions about excitatory and inhibitory synaptic mechanisms from simulations-based imaging data, diversity of injury types in the patient population and specific methods used.

Main Points that need to be addressed:

1. The conclusions from the modeling data linking gross MRI measures to excitatory and inhibitory synaptic activity as a categorical primary conclusion (inhibitory connection loss in CT+ sample) appears overstated since the imaging modalities used to develop the model cannot distinguish inhibitory and excitatory connections. The authors should acknowledge this limitation and include this caveat in the conclusions.

2. The authors need to discuss the of heterogeneity in the type of TBI these patients might have experienced. A more thorough characterization of injury severity is needed as the presence of positive acute imaging outcomes is consistent with behavioral outcomes associated with moderate rather than mild TBI. Integration of behavioral findings, rather than reference unpublished works in preparation, would strengthen the manuscript.

3. Specific methods used including those for voxel assignment, optimization, how the lesions in simulations in patients with lesions was handled, choice for brain parcellation based upon anatomical landmarks rather than using a functional atlas and steps taken to address motion artefact in resting-state fMRI need to be explained.

Detailed comments from the reviewers are included below.

Reviewer 1

The authors present a proof-of-principle study examining how computational modeling can be used to relate differences in modeling parameters and dynamics to TBI outcomes. They fit model parameters to data from mTBI patients (both with and without identified lesions) and compare the results to healthy individuals. They are able to identify certain modeling parameters, such as the strength of inhibitory connections, that correlate with behavioral measures post TBI. Interestingly, and somewhat unexpectedly, these correlations are found in the subgroup without the presence of lesions. In general, I think that this is a well-written and interesting paper that opens the door for future work in this area. Despite the somewhat small sample size, the authors are still able to show the utility of the approach, and I would only expect the findings to be more robust and of interest as larger data sets become available. I therefore recommend the article for publication after the following minor comments are addressed.

1. I am a bit confused by the paragraph starting in line 230. It appears that here, voxels are assigned to an ROI in a probabilistic fashion that allows the determination of weights. However, in my reading of the section describing the structural connectomes, I thought that voxels were assigned only to a single ROI. Please clarify - the procedure should be the same in both cases!

2. Please clarify the procedure for optimizing G and J_i (lines starting 317). For each value of G tested do you first optimize J_i before comparing with other G values? It seems like this would be necessary before choosing the optimal value of G. If not, please explain why.

3. In line 393, when you say a "global reduction in SC" does this refer to the total number of streamlines? Please clarify what you are actually measuring here.

4. In line 461 please remind the reader what a higher/lower score on the TBI symptoms test actually means in terms of performance. I had definitely forgotten by the time I read this and since this is driven by the negative patient group it can be confusing.

5. I think that in the discussion, it would be useful to discuss the large amount of heterogeneity in the type of TBI these patients might have experienced. From what I can tell, the only analysis of the type of TBI is related to the presence of a visible lesion. I completely understand that this is just a reality of the data - it is one of the reasons that it is hard to do studies on TBI patients. However, this also means that if you had a larger data set where patients could be grouped by the specific subtype/location of the injury, I would expect this approach to perform better. If you are looking for changes in model parameters in specific regions, but the actual injury in the patient population varies, this will limit the power of the approach. Again, I think this is just worthy of pointing out because in larger data sets used in future work, relationships might be more clear/statistically detectable.

Reviewer 2:

This is an intriguing paper using advanced simulation and brain imaging approaches to disentangle heterogeneous outcomes post injury. The authors focus their attention on the literature associated with mTBI. The methods are integrative and goals to document the nature of network response post injury are important. There are significant concerns however, with respect to achieving the stated goal in this specific sample and much clarification in the modeling is needed. Concerns are reported here in order of importance.

One critical conceptual issue for this paper is that the goal of the study is to advance the understanding of heterogeneous outcomes in mTBI, but the sample studied is not an mTBI sample. The positive CT scan present in 1/3 of the sample makes is clinically meaningful where acute positive CT findings represent an important distinction between mild and complicated mild (which hold similar outcomes to moderate injury) subtypes. Not too surprisingly the results fall along the lines between mild and moderate injuries. What would be more interesting is if the network results with regard to inhibitory control tracked behavioral outcome. The authors refer to other work (Good et al) that perhaps has taken this approach-- but no reference is available.

The exclusion of individuals with "previous significant TBI (LOC>5mins)" appears arbitrary given the inclusion of mild, and complicated mild/moderate injuries which have different clinical trajectories.

Given that so much of the goals for this paper relies upon the fit between the data and the simulated brain model, much greater detail regarding how gross measurements of water diffusion and blood flow in MRI methods can be reliably linked to excitatory/inhibitory networks is needed. The models being used are a distinct strength of the paper, but they also require continued validation in human networks (and clinical samples) so much greater information about how they are applied in this data set would increase confidence in the findings.

For example, the authors could provide very detailed linkage between MRI connectomics and parameters to GABA/NMDA synapses (Fig 1 but with much greater detail). Also, what are the indicators of relative success of the model linkage between simulation and human MRI data at the subject level? With regard to parameter fits, what are the confidence intervals and what is the threshold whereby simulations and acquired data cannot be mapped onto one another?

With regard to the TBI group with lesions, there needs to be more information about how those lesions were modeled in the VTB sims and how they were handled during processing and within the networks. The authors note no effect of lesions on the data - how was this established?

Choice of brain parcellation of 96 regions based upon anatomical landmarks needs justification. Why not a functional atlas (e.g., Power; Gordon) or data driven approach?

With regard to the rsfMRI, as the authors are likely aware, there is great concern about head motion in the literature. The authors chose not to use GSR, which is defensible, but they should provide a citation as to why this step was not taken and then also offer details regarding framewise displacement and how this was corrected, including a table with these data for between-group comparison.

## Author Response

Synthesis of Reviews:

Computational Neuroscience Model Code Accessibility Comments for Author (Required):

The study uses previously developed code that is available

Significance Statement Comments for Author (Required):

Caveats associated with conclusions about inhibitory connections based on imaging data needs to be acknowledged and/or conclusions tempered to reflect these limitation

Comments on the Visual Abstract for Author (Required):

N/A

Synthesis Statement for Author (Required):

The study examines how computational modeling of individualized brains using imaging data from patients can be used to relate differences in modeling parameters and dynamics to variable outcomes after brain injury. Overall, the reviewers are positive about the approach and the ability of the simulations to generate mechanistic predictions regarding connectivity. While this is a potentially useful approach, there are specific issues that need additional acknowledgement and /or clarification including the limitations associated with drawing conclusions about excitatory and inhibitory synaptic mechanisms from simulations-based imaging data, diversity of injury types in the patient population and specific methods used.

Main Points that need to be addressed:

1. The conclusions from the modeling data linking gross MRI measures to excitatory and inhibitory synaptic activity as a categorical primary conclusion (inhibitory connection loss in CT+ sample) appears overstated since the imaging modalities used to develop the model cannot distinguish inhibitory and excitatory connections. The authors should acknowledge this limitation and include this caveat in the conclusions.

2. The authors need to discuss the of heterogeneity in the type of TBI these patients might have experienced. A more thorough characterization of injury severity is needed as the presence of positive acute imaging outcomes is consistent with behavioral outcomes associated with moderate rather than mild TBI. Integration of behavioral findings, rather than reference unpublished works in preparation, would strengthen the manuscript.

3. Specific methods used including those for voxel assignment, optimization, how the lesions in simulations in patients with lesions was handled, choice for brain parcellation based upon anatomical landmarks rather than using a functional atlas and steps taken to address motion artefact in resting-state fMRI need to be explained.

Detailed comments from the reviewers are included below.

Reviewer 1

The authors present a proof-of-principle study examining how computational modeling can be used to relate differences in modeling parameters and dynamics to TBI outcomes. They fit model parameters to data from mTBI patients (both with and without identified lesions) and compare the results to healthy individuals. They are able to identify certain modeling parameters, such as the strength of inhibitory connections, that correlate with behavioral measures post TBI. Interestingly, and somewhat unexpectedly, these correlations are found in the subgroup without the presence of lesions. In general, I think that this is a well-written and interesting paper that opens the door for future work in this area. Despite the somewhat small sample size, the authors are still able to show the utility of the approach, and I would only expect the findings to be more robust and of interest as larger data sets become available. I therefore recommend the article for publication after the following minor comments are addressed.

1. I am a bit confused by the paragraph starting in line 230. It appears that here, voxels are assigned to an ROI in a probabilistic fashion that allows the determination of weights. However, in my reading of the section describing the structural connectomes, I thought that voxels were assigned only to a single ROI. Please clarify - the procedure should be the same in both cases!

In the case of the FC and FCD, voxels were weighted according to their location in the parcellation map (central values were weighted more heavily than voxels on the periphery). This was done to minimize potential partial volume bias. On the contrary, in the construction of the SC matrices, seed and target ROIs were white matter voxels adjacent to gray matter voxels, within each region of the RM-96 parcellation scheme.

We made changes to the section describing the dwMRI pre-processing and tractography to make this distinction more clear (lines 200-201).

2. Please clarify the procedure for optimizing G and J_i (lines starting 317). For each value of G tested do you first optimize J_i before comparing with other G values? It seems like this would be necessary before choosing the optimal value of G. If not, please explain why.

Yes, J_i was optimized via the FIC algorithm for every simulation (and therefore every value of G tested). We have clarified this in the section describing the Parameter Space Exploration (lines 316-317).

3. In line 393, when you say a "global reduction in SC" does this refer to the total number of streamlines? Please clarify what you are actually measuring here.

PLS found the CT/MRI positive patients had lower SC weights than CT/MRI negative patients in many voxels, therefore indicating a ’global’ effect. SC weights are the total number of streamlines connecting two regions divided by the total number of streamlines successfully sent from the seed mask as described in 214-227.

We have improved the wording of the section reporting the results from the group comparison PLS of SC weights to make this clearer (lines 408-412).

4. In line 461 please remind the reader what a higher/lower score on the TBI symptoms test actually means in terms of performance. I had definitely forgotten by the time I read this and since this is driven by the negative patient group it can be confusing.

We have added this reminder (lines 488-490)!

5. I think that in the discussion, it would be useful to discuss the large amount of heterogeneity in the type of TBI these patients might have experienced. From what I can tell, the only analysis of the type of TBI is related to the presence of a visible lesion. I completely understand that this is just a reality of the data - it is one of the reasons that it is hard to do studies on TBI patients. However, this also means that if you had a larger data set where patients could be grouped by the specific subtype/location of the injury, I would expect this approach to perform better. If you are looking for changes in model parameters in specific regions, but the actual injury in the patient population varies, this will limit the power of the approach. Again, I think this is just worthy of pointing out because in larger data sets used in future work, relationships might be more clear/statistically detectable.

We agree that with larger datasets, such as the full Track-TBI LONG sample, finer grouping of CT/MRI positive patients by lesion type and location of injury would be an excellent approach. Combined with the TVB approach there would be potential to explore lesion specific changes to local network dynamics, similar to previous work done in stroke patients (Aerts et al., 2018). We have added these ideas for future work to the discussion (lines 650-651).

Reviewer 2:

This is an intriguing paper using advanced simulation and brain imaging approaches to disentangle heterogeneous outcomes post injury. The authors focus their attention on the literature associated with mTBI. The methods are integrative and goals to document the nature of network response post injury are important. There are significant concerns however, with respect to achieving the stated goal in this specific sample and much clarification in the modeling is needed. Concerns are reported here in order of importance.

One critical conceptual issue for this paper is that the goal of the study is to advance the understanding of heterogeneous outcomes in mTBI, but the sample studied is not an mTBI sample. The positive CT scan present in 1/3 of the sample makes is clinically meaningful where acute positive CT findings represent an important distinction between mild and complicated mild (which hold similar outcomes to moderate injury) subtypes. Not too surprisingly the results fall along the lines between mild and moderate injuries. What would be more interesting is if the network results with regard to inhibitory control tracked behavioral outcome. The authors refer to other work (Good et al) that perhaps has taken this approach-- but no reference is available.

This point is noted and we have added some dialogue to the Discussion about the heterogeneity in the severity of injury within our sample. We do note that in this work the CT/MRI positive group was defined by a positive finding on CT or MRI, and as MRI is generally considered more sensitive to TBI than CT this is an important distinction from previous studies that have considered complicated mTBI solely on the basis of a positive CT scan (Iverson et al., 2012; Voormolen et al., 2019). Additionally, we did in fact show that our measure of inhibitory connection strengths tracked behavioural outcomes at six months (Figure 7), an effect that was primarily driven by the CT/MRI negative group (Figure 8, A-C).

We have removed the references to other unpublished work (Good et al, in prep A and B).

Changes implemented in lines 605-616.

The exclusion of individuals with "previous significant TBI (LOC>5mins)" appears arbitrary given the inclusion of mild, and complicated mild/moderate injuries which have different clinical trajectories.

Individuals with previous significant TBI (defined by LOC>5mins) were excluded as it is well established that repeated TBI increases the likelihood of prolonged symptomology and potentially long-term neurodegeneration (Guskiewicz et al., 2005; Prins, Alexander, Giza, & Hovda, 2013; Slobounov, Sebastianelli, Cao, & Newell, 2007). While we included "uncomplicated" mTBI patients as well as those with more "complicated" mTBI (CT/MRI positive), we assessed differences between these two groups. Unfortunately, we lacked the data to consider the repeated TBI as an independent variable as well.

We also note that our inclusion and exclusion criteria have precedent in this sample, as previous work studying the white matter integrity (Yuh et al., 2014) and resting-state functional connectivity (Palacios et al., 2017) of these patients used the same inclusion/exclusions criteria. Our use of similar inclusion/exclusion criteria allows for easier comparison with these studies.

Given that so much of the goals for this paper relies upon the fit between the data and the simulated brain model, much greater detail regarding how gross measurements of water diffusion and blood flow in MRI methods can be reliably linked to excitatory/inhibitory networks is needed. The models being used are a distinct strength of the paper, but they also require continued validation in human networks (and clinical samples) so much greater information about how they are applied in this data set would increase confidence in the findings.

Large-scale models of brain activity, such as the TVB approach taken in this paper, are increasingly being used to in cognitive and clinical neuroscience (for review: Breakspear, 2017). In these large-scale models, neural populations are represented by mean field models that are reductions of spiking models (Deco et al., 2014). Neural populations have robust biophysical features, such as steady state and periodic behaviour, which are reliably captured by mean field models (Sanz-Leon, Knock, Spiegler, & Jirsa, 2014; Schirner, Mcintosh, Jirsa, & Deco, 2018). The mean field approximations that we use have been validated independently (Gustavo Deco & Hugues, 2012). The output from the mean field model is then modeled with a hemodynamic response function for comparison to resting-state fMRI data (Sanz-Leon et al., 2014). This approach has been taken previously to model biophysical phenomena across a number of groups, from healthy adults (Jirsa, Sporns, Breakspear, Deco, & Mcintosh, 2010; Roy et al., 2014), to stroke (Falcon et al., 2016, 2015), tumour (Aerts et al., 2018), and dementia (Zimmermann et al., 2018). Specifically, Zimmermann et al (2018) showed proof-of-concept that these models can exhibit inhibitory/excitatory imbalance that is known to occur in clinical populations and corroborates animal models of Alzheimer’s disease.

Lastly, we note that modeling the collective behaviour of neurons rather than individual neurons themselves is not a limitation. Cognitive states, perception, and movement all arise from the collective behaviour of cortical, thalamic, and spinal neurons rather than the individual spiking of single neurons (Fries, 2005; Hoel, Albantakis, & Tononi, 2013; Kumar, Rotter, & Aertsen, 2010; Montijn, Meijer, Lansink, & Pennartz, 2016), while neural modeling studies have shown that macroscopic features may be more deterministic than microscopic features for behaviour of the system. This all suggests that studying the collective behaviour of neurons is conducive to understanding behaviour, much in the same way that an epidemiologist might study populations of people to understand an epidemic.

To ensure clarity, we made minor modifications to the ’The Virtual Brain’ heading of the Methods section and added two citations, line 271-272.

For example, the authors could provide very detailed linkage between MRI connectomics and parameters to GABA/NMDA synapses (Fig 1 but with much greater detail). Also, what are the indicators of relative success of the model linkage between simulation and human MRI data at the subject level? With regard to parameter fits, what are the confidence intervals and what is the threshold whereby simulations and acquired data cannot be mapped onto one another?

The empirical and simulated FC and FCD matrices of every participant were inspected qualitatively to ensure they showed adequate fit. Features that would have eliminated subjects were homogenous looking FC matrices without the usual ’blocky’ pattern of regions with higher and lower correlations, single regions with very low correlations with all other regions on the FC, and a lack of variability in correlations across time in the FCD matrix. We did not eliminate any subjects based on these checks.

We also checked for outliers in fit of the FC and FCD matrices by converting the uncentered correlations and Kolmogorov-Smirnov distances to z-score and screening for any participants with an absolute value greater than 3. Again, we identified no subjects with outlier fit on either measure by this criteria.

Lastly, we ran 20 iterations of each value of G explored for each subject to account for the effect of intra-subject variability due to our stochastic model. We selected the value of G that was the best-fitting most commonly (mode) across all iterations (Figure 4B).

For more information on the fitting procedure, we would refer the reader to these foundational TVB papers (Ritter, Schirner, McIntosh, & Jirsa, 2013; Sanz Leon et al., 2013; Schirner et al., 2018).

We’ve added notes describing these steps to the Results section, lines 431-436.

With regard to the TBI group with lesions, there needs to be more information about how those lesions were modeled in the TVB sims and how they were handled during processing and within the networks. The authors note no effect of lesions on the data - how was this established?

Individual tracts from the participants in the CT/MRI positive group were checked for unrealistic characteristics that would indicate a problem with the tractography. We did not identify any problem tracts through this procedure. This was expected, as the radiological findings defining the CT/MRI positive group tended to be subtle (see the newly added Table 2, lines 853).

Choice of brain parcellation of 96 regions based upon anatomical landmarks needs justification. Why not a functional atlas (e.g., Power; Gordon) or data driven approach?

The RM-96 parcellation was developed to harmonize cytoarchitectonic, topographic, and functional definitions of brain regions across primate species (Kotter & Wanke, 2005). In computational work where functional and structural brain imaging data are integrated within a single model, such as this manuscript, the use of a parcellation scheme developed with multiple data types in mind is a distinct advantage. Indeed, the RM-96 parcellation has been used successfully in conjunction with TVB models (Ritter et al., 2013; Shen et al., 2019) in non-clinical populations previously. Furthermore, Shen et al (2019) validates the RM-96 parcellation with gold standard tract tracing data as well as diffusion-based tractography in the macaque, while also demonstrating how the RM-96 parcellation could be used in whole-brain network model simulations similar to those used in this manuscript, using the RM-96 parcellation.

We have added a note to this effect on lines 187-191.

With regard to the rsfMRI, as the authors are likely aware, there is great concern about head motion in the literature. The authors chose not to use GSR, which is defensible, but they should provide a citation as to why this step was not taken and then also offer details regarding framewise displacement and how this was corrected, including a table with these data for between-group comparison.

A limitation of our study is that we used a single pre-processing strategy. While global signal regression may reduce the relationship between head motion and FC, it can also result in distance dependent artefacts in FC unless used in combination with censoring methods (Ciric et al., 2017). Future work would benefit from employing multiple pre-processing strategies to bolster the robustness of findings.

No participants were removed due to head motion (absolute head motion at a single time point was no greater than 3.5 mm and relative head motion did not exceed 2.5 mm for any participants. A one-way ANOVA showed the comparison participants (M=0.27, SD=0.28), CT/MRI positive patients (M=0.25, SD=0.20), and CT/MRI negative patients (M=0.23, SD=0.17) did not differ in mean absolute head motion, F(2,77)=0.22, p=0.801. Similarly the comparison participants (M=0.06, SD=0.03), CT/MRI positive (M=0.07, SD=0.02), and CT/MRI negative patients (M=0.10, SD=0.09) did not differ in mean relative head motion, F(2,77)=2.00, p=0.14.

We have added this information to the methods section, lines 248-255.
